# Nickel treatment of soybean seeds: evaluating optimal levels for *Bradyrhizobium* spp. survival, nitrogen fixation, physiological traits and grain yield

**DOI:** 10.3389/fpls.2025.1656956

**Published:** 2026-01-12

**Authors:** Luiz Gustavo Moretti, Carlos Alexandre Costa Crusciol, Marco Antonio Nogueira, João William Bossolani, José Roberto Portugal, Mariangela Hungria

**Affiliations:** 1 Department of Crop Science, College of Agricultural Sciences, São Paulo State University (UNESP), Botucatu, SP, Brazil; 2 Embrapa Soja, Londrina, PR, Brazil

**Keywords:** acetylene reduction activity, biological nitrogen fixation, *Glycine max* (L.) Merrill, micronutrient, nodulation

## Abstract

**Introduction:**

Accumulating evidence indicates that fertilizing soybean with nickel (Ni) can enhance biological nitrogen fixation (BNF) and plant productivity. Seed application is ideal for promoting nodule formation and early plant development, but this practice raises the risk of toxicity by reducing the difference between beneficial and harmful doses. Unfortunately, studies of the effects of Ni on the survival of *Bradyrhizobium* spp. applied as inoculants or on BNF have not yielded a consensus on an optimal dose.

**Methods:**

The objective of this study was to establish Ni thresholds that maximize physiological and productivity benefits for soybean, balancing Ni’s positive effects on BNF and plant growth against risks of phytotoxicity and bacterial inhibition. Soybean seeds were treated with nickel sulfate (NiSO_4_·6H_2_O) at six doses: 0, 60, 120, 180, 240, and 300 mg Ni kg^−1^. We then assessed the effects of seed treatment with Ni on the recovery of *Bradyrhizobium* cells from treated seeds, BNF as assessed by continuous-flow analysis of acetylene reduction activity (ARA), and soil CO_2_ evolution in greenhouse experiments. In addition, we evaluated the impact of Ni dose on the physiological, nutritional, agronomic traits, and grain yield of soybean in multi-site field trials over two cropping seasons.

**Results and Discussion:**

Ni doses of up to 60 mg kg^−1^ enhanced nitrogenase activity, nodulation, nodule biomass, and grain yield without compromising *Bradyrhizobium* viability. Doses exceeding this threshold reduced bacterial survival, nodulation, and yield, indicating Ni toxicity. The field trials exhibited a natural gradient in soil Ni levels and texture (0.4–0.6 mg dm^−3^ sandy to clayey), which helps explain differences in response magnitude and reinforces the need for contextualized recommendations. Applying a second-order polynomial regression to mean standardized Z-scores of integrated agronomic traits revealed a significant quadratic response (*p* < 0.05). Consequently, an agronomically optimal range of 50–100 mg Ni kg^−1^ is recommended to sustainably optimize soybean growth and N fixation by balancing the benefits and risks of Ni application.

## Highlights

- Treating soybean seeds with low Ni doses (≤ 120 mg kg^-1^ seed) increases soil CO_2_ efflux, indicating elevated microbial activity.
- Excessive Ni application (> 120 mg kg^-1^ seed) induces toxicity, impairing *Bradyrhizobium* viability and reducing nodulation.
- Nitrogenase activity peaks between 0 and 60 mg Ni kg^-1^, significantly enhancing carbon and nitrogen metabolism.
- Optimal Ni range (50–100 mg kg^-1^ seed) enhances biological nitrogen fixation (BNF) and improve soybean grain yield.

## Introduction

In tropical regions, the primary nitrogen (N) source for soybean (*Glycine max* (L.) Merrill) is biological nitrogen fixation (BNF) performed by *Bradyrhizobium* spp., which form symbiotic relationships with soybean roots (Kutman et al., 2014; Moretti et al., 2020b, 2024). BNF eliminates the need for synthetic N-fertilizers (Moretti et al., 2018), contributing to an estimated annual economic benefit of approximately 15 billion USD in Brazil alone (Hungria and Mendes, 2015; Moretti et al., 2020b). Consequently, agronomic practices that can enhance BNF and plant N uptake are highly desirable.

A vital enzyme in plant N-metabolism is urease, which requires the essential micronutrient nickel (Ni) for its activation (Brown et al., 1987; Fabiano et al., 2015). Ni also positively impacts BNF. Because of Ni’s ability to enhance various physiological processes and improve crop yields, Ni application is an increasingly common agronomic practice (Freitas et al., 2018, 2019). However, high concentrations of Ni can inhibit enzyme function, disrupt cellular processes, and cause visible symptoms of toxicity, such as chlorosis (yellowing of leaves) and necrosis (death of plant tissue) (Chen et al., 2009). To avoid phytotoxicity, the application of Ni must be carefully managed (Barcelos et al., 2017; Oliveira et al., 2022a, 2022b) by optimizing the conditions of application, including both the dose and method (Rodak et al., 2024).

Soil available Ni concentrations and its availability to plants may be influenced by textural variation, from sandy to clayey soils, which is justified by the well-established role of clay and organic matter fractions in Ni adsorption, retention, and buffering in soils (Rodak et al., 2024). Ni absorption by plants varies depending on the method of application, which also affects nutrient use efficiency, yield, and agricultural profitability (Macedo et al., 2016; Rodak et al., 2024). When supplying micronutrients via the soil, high doses may be needed to counteract losses by leaching, erosion, and immobilization (Levy et al., 2019). Although this can create residual effects for subsequent crops (Freitas et al., 2018), in the medium and long term, metals may accumulate in the trophic system (Rodak et al., 2022). Nutrient application by leaf spraying demands lower doses than soil application to prevent toxicity, but the need for several applications during the crop cycle to reach an adequate dose may increase production costs. Moreover, the ability of leaf spraying to provide nutrients at the early stages of plant development is limited (Niu et al., 2021).

Treating seeds with micronutrients not only reduces the required dose (and thus fertilizer costs) but also promotes plant nutrition in the early plant growth stage, when the poorly developed root system limits the absorption of nutrients from the soil (Farooq et al., 2012). However, seed treatment can cause phytotoxicity to seedlings, especially when micronutrients are applied, because the difference between appropriate and toxic doses is small (Lavres et al., 2016). Moreover, elevated Ni levels can inhibit *Bradyrhizobium* spp. survival and function, undermining BNF (Chaintreuil et al., 2007). Therefore, there is a paradox in which the element intended to enhance BNF ultimately impairs the bacteria essential for this process.

Understanding the delicate balance required to achieve the benefits of Ni seed application is essential for advancing agronomic practices. Although interest in Ni fertilization has increased, the specific dose that optimizes plant benefits while avoiding phytotoxicity and negative effects on *Bradyrhizobium* spp. remains uncertain. Thus, the aim of this study was to understand this balance and establish the optimal Ni range by conducting experiments under controlled conditions and in the field. In determining threshold Ni doses that maximize both physiological and productive benefits, our focus was on balancing the positive effects of Ni on BNF and plant growth against the potential risks of phytotoxicity and bacterial inhibition.

## Materials and methods

### *Experiment I* - recovery of *Bradyrhizobium* cells from soybean seeds

To assess the recovery of *Bradyrhizobium* cells from soybean seeds, a controlled experiment was carried out in the Soil Biotechnology Laboratory at Embrapa Soja in Londrina, Paraná (PR), Brazil, under controlled conditions in a completely randomized design with eight replicates. The strain *Bradyrhizobium japonicum* SEMIA 5079 (=CPAC 15, =CNPSo 07), which is used in commercial inoculants for soybean cultivation in Brazil, was used. The strain is deposited at the “Diazotrophic and Plant Growth Promoting Bacteria Culture Collection of Embrapa Soja (WFCC Collection # 1213, WDCM Collection # 1054).

Seeds were treated with one of six doses of nickel sulfate (NiSO_4_.6H_2_O) (0, 60, 120, 180, 240, and 300 mg Ni kg seeds^-1^) corresponding to 0, 3, 6, 9, 12, and 15 g Ni ha^−1^. The seeds were then inoculated with a high bacterial load of 7 × 10^9^ cells mL^−1^ to provide 1.2 × 10^6^ cells per seed. Three biological samples were transferred to sterile Erlenmeyer flasks containing 100 mL of sterile saline solution (0.85%) supplemented with Tween 80 (0.4 mL L^-1^). Each sample contained 100 seeds treated with NiSO_4_·6H_2_O. The flasks were subjected to horizontal shaking at 150 rpm for 20 min, and the recovered saline solution was referred to as the recovery sample (Pinto et al., 2023).

The recovery sample was serially diluted (10^-1^, 10^-2^, 10^-3^, and 10^-4^), and aliquots of 100 µL of each dilution were spread onto Petri dishes containing modified-yeast mannitol agar (YMA) culture medium (Hungria et al., 2017). The medium was supplemented with 10% Congo Red solution (0.25%), actidione (667 μL L^-1^ cycloheximide [84 μg mL^-1^ in ethanol]), and vancomycin (333 µL L^-1^ vancomycin hydrochloride [0.3 g mL^-1^]) to inhibit soil or seed contaminants (Rodrigues et al., 2020). The plates were incubated for seven days in the dark at 28 ± 2°C. Colony-forming units (CFUs) were then quantified on each plate and expressed as CFUs per seed (CFU seed^-1^). Based on the results, recovery was classified as low (up to 100 CFUs per 100 µL of recovery sample), medium (between 100 and 200 CFUs per 100 µL of recovery sample), or high (above 300 CFUs per 100 µL of recovery sample).

### *Experiment II*- evaluation of biological nitrogen fixation based on acetylene reduction activity

To measure BNF activity via the acetylene reduction activity (ARA) method using the *in situ* continuous flow protocol (Devi et al., 2010), a controlled experiment was conducted in a greenhouse at Embrapa Soja, Londrina, PR, Brazil, under greenhouse conditions in a completely randomized design with eight replicates. The soil, which was classified as a Ferralsol according to the International System (Soil Survey Staff, 2014), had the following chemical properties: pH(_CaCl2_) 5.1; calcium (Ca) 26 mmol_c_ dm^-3^; magnesium (Mg) 5 mmol_c_ dm^-3^; aluminum (Al) 0 mmol_c_ dm^-3^; H^+^+ Al 67 mmolc dm^-3^; potassium (K) 5.8 mmol dm^-3^; phosphorus (P) (resin) 16 mg dm^-3^; sulfur (S) 4.8 mg dm^-3^; boron (B) 0.36 mg dm^-3^; copper (Cu) 0.9 mg dm^-3^; iron (Fe) 49 mg dm^-3^; manganese (Mn) 9.5 mg dm^-3^; zinc (Zn) 2.7 mg dm^-3^; organic matter 20 g dm^-3^; Ni 0.2 mg dm^-3^; cation exchange capacity (CEC) 99 mmol_c_ dm^-3^. The physical properties of the soil were clay 600 g dm^-3^; silt 50 g dm^-3^; and sand 350 g dm^-3^. The liming requirement was determined using the base saturation method to reach 70% of CEC (van Raij. et al., 1997).

The same strain and treatments used in *Experiment I* were used in *Experiment II*. Soybean seeds (cultivar BRS 1001 IPRO) were inoculated with a high bacterial load of 7 × 10^9^ cells mL^-1^ to provide 1.2 × 10^6^ cells seed^-1^. Three seeds were sown in a polyvinyl chloride (PVC) pot (15 cm in diameter and 30 cm in height) filled with the clay soil described above. The pots were housed under controlled irrigation conditions in a greenhouse with the temperature regulated to maintain 28°C both day and night. Each pot contained 2 kg of soil, and the irrigation frequency was determined based on the soil water retention curve, maintaining soil moisture at 70–90% of field capacity. In addition, sterilized N-free standard nutrient solution was applied (De Oliveira-Francesquini et al., 2017). After one week, the seedlings were thinned to one plant per pot, six replicates were performed per treatment.

**Figure 1:**
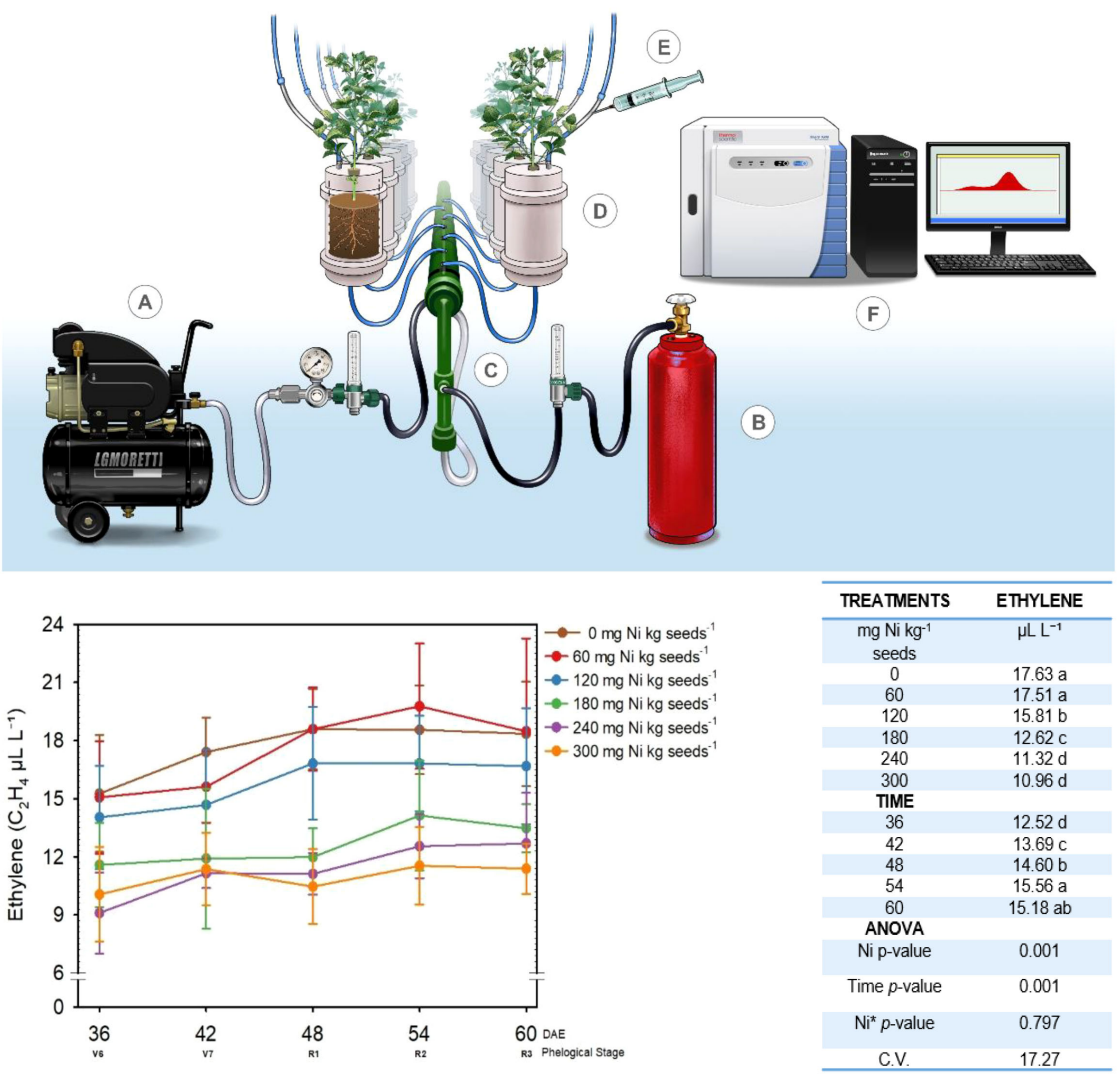
Simplified diagram of the device used to monitor acetylene reduction activity (ARA) and its operational procedures and Acetylene reduction activity in nodulated soybean as a function of the Ni dose applied to seeds. llustration of an experimental setup involves equipment marked A to F, including an air compressor **(A)**, a gas cylinder **(B)**, connectors **(C)**, plant containers **(D)**, a syringe **(E)**, and a gas chromatograph connected to a computer **(F)**. The ethylene concentration (µL L^−1^) at different phenological stages (days after emergence, DAE) is shown Londrina, Paraná, Brazil. The averages of 200 readings per phenological stage (days after emergence, DAE) are shown. Means followed by different letters are significantly different by the LSD test at *p* < 0.10.

ARA was measured during phenological stages V_6_ – R_3_ (Fehr et al., 1977) as illustrated in **Figure 1**. On the day before sampling, all pots were watered thoroughly and left to drain overnight. The next morning, a lid was sealed around the base of each plant, and an acetylene:air mixture (1:9) was introduced into the pots through an inlet at the bottom at a flow rate of 1 L min^-1^ (**Figures 1A, B**). When the steady state was reached after 15 min of flow (**Figures 1C, D**), three gas samples (1 mL each) were collected from the outlet of each pot using syringes (**Figure 1E**). Once all gas samples were collected, acetylene-free air was flushed through all pots for 1 h to remove any remaining acetylene. Flushing the pots with air ensured that the plants were exposed to acetylene for only a very short period each day.

The concentration of ethylene (C_2_H_4_) in the gas samples was determined using a gas chromatograph equipped with a flame ionization detector (FID) (Thermo Scientific; TRACE™ 1310; Waltham, MA) (**Figure 1F**). The run time to measure the ethylene concentration was 1 min, and the concentrations were expressed in µL L^-1^. All details, including the carrier gas, flow rate, and temperatures, as well as whether concentrations were determined with reference to a calibration curve, are described by Devi et al. (2014).

### *Experiment III*- measurement of carbon dioxide in soil and plant growth parameters

Microbial activity and root respiration were assessed by monitoring carbon dioxide (CO_2_) in the soil using an Arduino prototype. The experiment was conducted at Embrapa Soja, Londrina, PR, Brazil (27° 16’ 60’’ S, 50° 35’ 7’’ W) under greenhouse conditions in a completely randomized design with eight replicates. *Experiment III* followed the standard protocol used in *Experiment II* with respect to controlled greenhouse conditions, soil attributes, cultivar, strains, and treatments.

**Figure 2:**
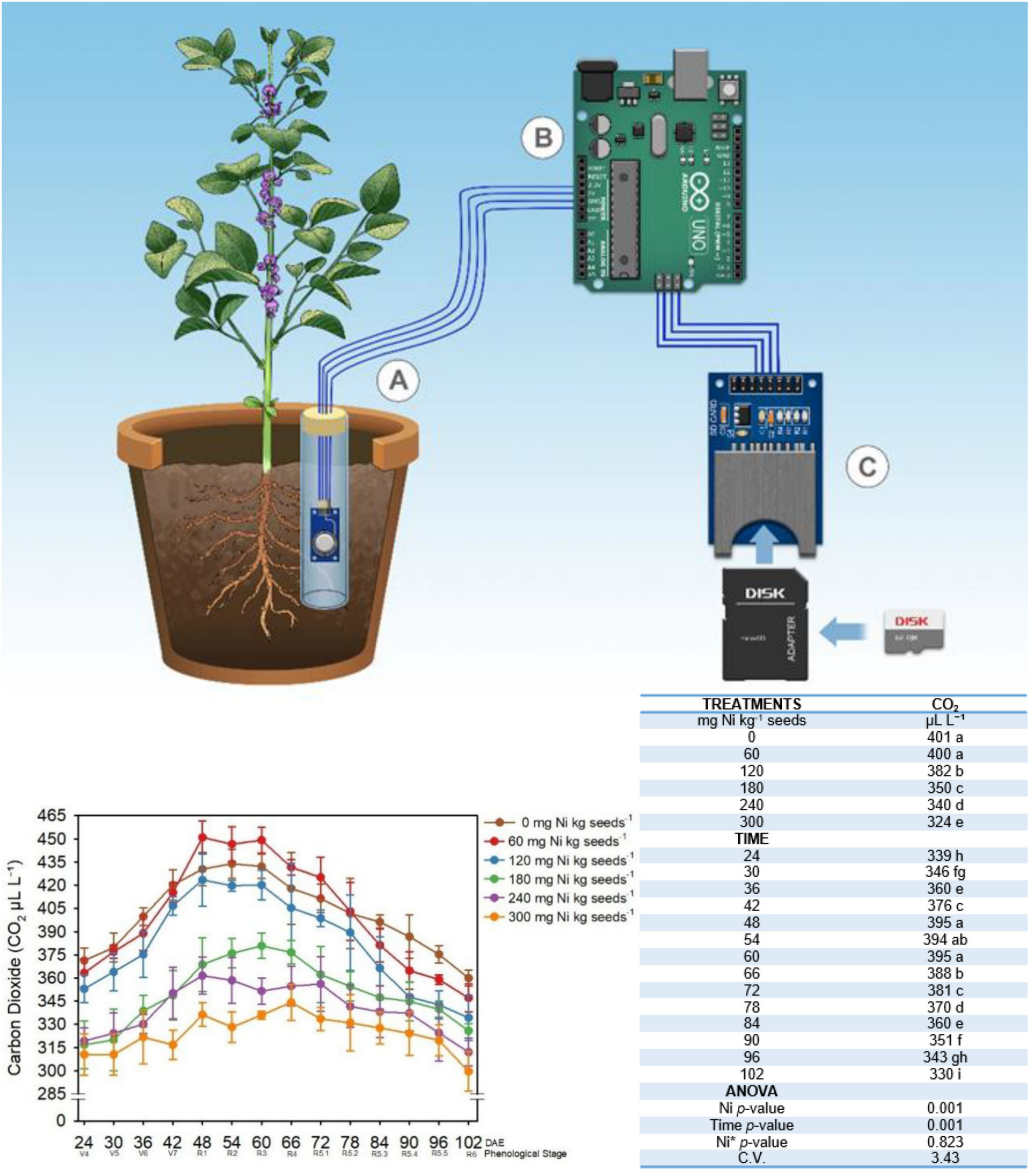
Simplified diagram of the measurement of CO_2_ using the Arduino prototype and its operational procedures. llustration of a plant in a pot with a connected sensor. The sensor **(A)** is linked to an Arduino board **(B)**, which in turn connects to an SD card module **(C)**. Londrina, Paraná, Brazil. CO_2_ concentration (µL L^−1^) in soil cultivated with soybean as a function of Ni dose in seed treatment. The averages of 200 readings per phenological stage (days after emergence, DAE) are shown. Means followed by different letters are significantly different by the LSD test at *p* < 0.10.

**Figure 2** presents a simplified diagram of the Arduino Uno R3 (ATmega32) prototype, which comprised an SD module, sensor, LCD display, power supply, USB cable, protoboard and its use. Code was uploaded via an Arduino IDE. Sensor data were processed, displayed, and recorded on an SD card. The prototype was placed in a sealed tube to protect against water, secured to prevent cable disconnection, and connected to a stabilized electrical network. The full methodology is detailed in Kormann et al. (2024). The Arduino prototype monitoring system was installed by inserting a 15-cm-long, 27-mm-diameter PVC tube into the top 12.5 cm of the soil, leaving 2.5 cm of the tube above the surface. The tube was positioned approximately 5 cm from the pot center, where soybean seeds were later planted. The tube’s bottom was sealed with Parafilm™ to prevent water from entering and damaging the sensor. An MQ-135 sensor was placed 12 cm below the soil surface inside the tube, which was then covered with Parafilm™ to block water entry during irrigation. The soil was prepared to prevent compaction around the tube and ensure that gas concentrations could be measured through the tube’s base.

The Arduino prototype was used to record CO_2_ every hour throughout the soybean cycle. The data were then used to calculate the average daily concentration of CO_2_ in the soil air. All details of validation or calibration are described by Kormann et al. (2024).

### *Experiment IV-* multi-site field trials

To evaluate the effects of Ni application on soybean nutritional, physiological, and production components and grain yield, field trials were conducted over two growing seasons (2022/2023 and 2023/2024) at three experimental sites in distinct regions of Brazil: (*I*) Carolina Farm in the municipality of Sengés, Paraná State (PR), southern Brazil; (*II*) the Lageado Experimental Farm, College of Agricultural Sciences (UNESP/FCA), Botucatu, São Paulo State (SP), southeastern Brazil; and (*III*) the Experimental Farm of the School of Engineering, Campus Ilha Solteira (UNESP/FEIS), Selvíria, Mato Grosso do Sul State (MS), central-western Brazil, all of them in a completely randomized design with eight replicates. **Supplementary Table 1** provides comprehensive experimental information for the three sites. Prior to sowing, the soybean seeds were treated with one of six doses of nickel sulfate (NiSO_4_.6H_2_O) corresponding to 0, 60, 120, 180, 240, and 300 mg Ni kg^-1^ and were then inoculated with a high bacterial load of 7 × 10^9^ cells mL^-1^ to provide 1.2 × 10^6^ cells per seed. Dolomitic lime (28% calcium oxide—CaO, 18% magnesium oxide—MgO, and 81% calcium carbonate equivalent—%ECaCO_3_) was applied 60 days before the experiments to increase base saturation in the topsoil (0.00–0.20 m) to 70%, following the procedure outlined by Cantarella et al. (2022).

**Supplementary Table 2** details the physical, chemical, and biological properties of the soil at a depth of 0.00–0.20 m, as well as the climate classification at each site according to the Köppen-Geiger system (Alvares et al., 2013). Physical attributes were determined following the methodology described by Donagema et al. (2017), and chemical properties were analyzed according to van Raij. et al. (1997). The autochthonous bacterial population capable of nodulating soybean was estimated using the most probable number (MPN) method with soybean plants, as described by O’Hara et al. (2016). Climatological data recorded during the experiments are shown in **Supplementary Figure 1**.

### Nutritional status

The nutritional status of the plants—based on leaf concentrations of N, P, K, Ca, Mg, S, Cu, Fe, Zn, Mn, and Ni—was assessed across the different treatments. Diagnostic leaves were collected at the R_2_ phenological stage (full flowering). Specifically, the third fully expanded trifoliate leaf from the apex downward was sampled, properly labeled, and dried in a forced-air circulation oven. The dried material was ground using a Wiley-type mill and stored in labeled plastic bags for nutrient analysis (AOAC, 2019).

### Photosynthetic parameters – gas exchange

Photosynthetic performance was assessed through non-destructive diagnostic leaf measurements at the R_2_ stage using a portable gas exchange system (CIRAS-3, PP Systems Inc., Amesbury, MA, USA). Instrument settings were standardized to 380–400 μmol mol^-1^ atmospheric CO_2_, photosynthetically active radiation (PAR) of 1,100 μmol photons m^-2^ s^-1^ (provided by LED light), chamber temperature between 25°C and 27°C, and relative humidity between 60% and 70%. A minimum equilibration period of 3 min was used for each measurement. All measurements were performed between 10:00 a.m. and 12:00 p.m. The following parameters were recorded: net photosynthetic rate (A; μmol CO_2_ m^-2^ s^-1^); stomatal conductance (gs; mol H_2_O m^-2^ s^-1^); intercellular CO_2_ concentration (Ci; μmol mol^-1^); transpiration rate (E; mmol H_2_O m^-2^ s^-1^); water use efficiency (WUE; μmol CO_2_ mmol^-1^ H_2_O), calculated as the A/E ratio; and carboxylation efficiency, calculated as the A/Ci ratio.

### Urease activity (EC 3.5.1.5)

Urease activity was assessed *in vivo* (Hogan et al., 1983) in diagnostic leaves at the R_2_ stage (Fehr et al., 1977). Discs of fresh tissue (100 mg) were incubated for 3 h in test tubes containing 8 mL of 50 mM phosphate buffer (pH 7.4), 0.2 M urea, and 0.6 M n-propanol. After incubation, 0.5 mL of the supernatant was added to 2.5 mL of Reagent I (0.1 M phenol and 170 μM sodium nitroprusside), which was followed by the addition of 2.5 mL of Reagent II (0.125 M NaOH, 0.15 M Na_2_HPO_4_·12H_2_O, and 3% NaOCl). The reaction was conducted in sealed tubes under constant agitation in a water bath at 37°C for 35 min. The ammonium concentration was measured spectrophotometrically at 625 nm using an NH_4_Cl standard curve. Urease activity was expressed as μmol N–NH_4_^+^ g^-1^ fresh weight h^-1^.

### Nitrate reductase activity (EC 1.7.1.1)

Nitrate reductase (NR) activity (Hewitt and Nicholas, 1964) was measured in diagnostic leaves at the R_2_ stage (Fehr et al., 1977). The leaves were ground in liquid nitrogen with a 1:2 ratio (g:mL) of extraction buffer composed of 25 mM Tris-HCl (pH 8.5), 1 M EDTA, 1 mM DTT, 1% BSA, 20 μM FAD, and 200 mM leupeptin. After centrifugation at 14,000 × g for 10 min at 4°C, 200 μL of the supernatant was added to the reaction buffer to a final volume of 0.5 mL. The reaction buffer contained 50 mM HEPES-KOH (pH 7.6), 10 mM MgCl_2_ (for active NR) or 5 mM EDTA (for total NR), 10 μM FAD, 3% casein, and 1 mM DTT. For total NR activity, the extract was pre-incubated for 10 min with 11 μL of a solution containing 250 mM AMP and 500 mM EDTA. The reaction was initiated by adding 25 μL of 5 mM NADH diluted in 100 mM potassium phosphate buffer (pH 7.0) and incubated for 30 min. The reaction was stopped with 62.5 μL of 500 mM zinc acetate. The solution was centrifuged, and the supernatant was used to determine nitrite concentration by colorimetric analysis (Hageman and Reed, 1980).

### RuBisCO activity (EC 4.1.1.39)

Ribulose-1,5-bisphosphate carboxylase/oxygenase (RuBisCO) activity (Reid et al., 1997) was measured in diagnostic leaves at the R_2_ stage (Fehr et al., 1977). Activity was calculated based on the change in absorbance between 0 and 1 min and expressed as μmol min^−1^ mg^−1^ protein.

### Plant development, nodulation, and N-ureides concentration

At the full flowering stage (R_2_), five plants per plot were sampled for evaluation of nodulation and root and shoot dry mass. The ureide-N content in petioles plus leaves was determined (Hungria, 1994) by colorimetric quantification (Herridge et al., 2022). To measure the total ureide concentration (allantoin and allantoic acid) as a BNF indicator, a 300-μL aliquot was incubated with 500 μL of Solution 1 (50% [v/v] 0.5 N NaOH and 50% [v/v] 0.15 N HCl) at 100°C for 5 min and subsequently cooled to room temperature. The mixture was then combined with Solution 2 (11.5% [v/v] 0.4 M phosphate buffer at pH 7, 11.5% [v/v] phenylhydrazine, 70% [v/v] 0.65 N HCl at −20°C, and 7% [v/v] potassium ferricyanide). The ureide-N concentration was determined colorimetrically at 535 nm.

### Agronomic parameters and grain yield

In both growing seasons (2021/22 and 2022/23), plants at the R_2_ stage (Fehr et al., 1977), were harvested and separated into shoots and roots. The roots were washed to remove substrate particles. Nodules were removed from the roots, oven-dried at 65°C for 72 h, counted, and weighed. At harvest, the 100-grain weight and grain yield were determined. For grain yield, 2-m lengths of four rows were harvested (1.8 m²), threshed and weighed. The weight was adjusted to 13% moisture and then converted to kilograms per hectare. To determine the 100-grain weight, 10 samples of 100 grains were weighed, and the results were adjusted to 13% moisture.

### Statistical analysis

The greenhouse experiments were arranged in a completely randomized design, whereas the field trials followed a randomized complete block design. Prior to analysis, data were tested for normality (Shapiro and Wilk, 1965) and homogeneity of variances (Levene, 1960). Upon confirmation of these assumptions, data were subjected to one-way analysis of variance (ANOVA) with the F-test applied at the 10% significance level. When significant differences were detected, data were compared using the least significant difference (LSD) test with a significance level of 10%. The agronomic variables were standardized through Z-score transformation in order to ensure comparability across different measurement scales. The standardized values (Z-scores) were computed by subtracting the mean and dividing by the standard deviation of each variable (only to agronomic assessments). Subsequently, a mean Z-score was calculated for each treatment group by averaging the Z-scores of all variables included in the analysis. This mean Z-score served as the response variable in a multiple polynomial regression model, with Ni doses applied through seed treatment as the explanatory variable.

## Results

### *Bradyrhizobium* viability response to nickel seed treatment

To assess the effects of treating soybean seeds with Ni on the viability of *Bradyrhizobium*, soybean seeds were treated with one of six doses of Ni prior to inoculation with a high dose (1.2 × 10^6^ cells seed^-1^) of *Bradyrhizobium*: 0, 60, 120, 180, 240, or 300 mg of Ni per kg of seed (mg kg^−1^ seed) (*Experiment I*). After incubation for 20 min, the recovery of viable *Bradyrhizobium* from the seeds was determined.

**Figure 3:**
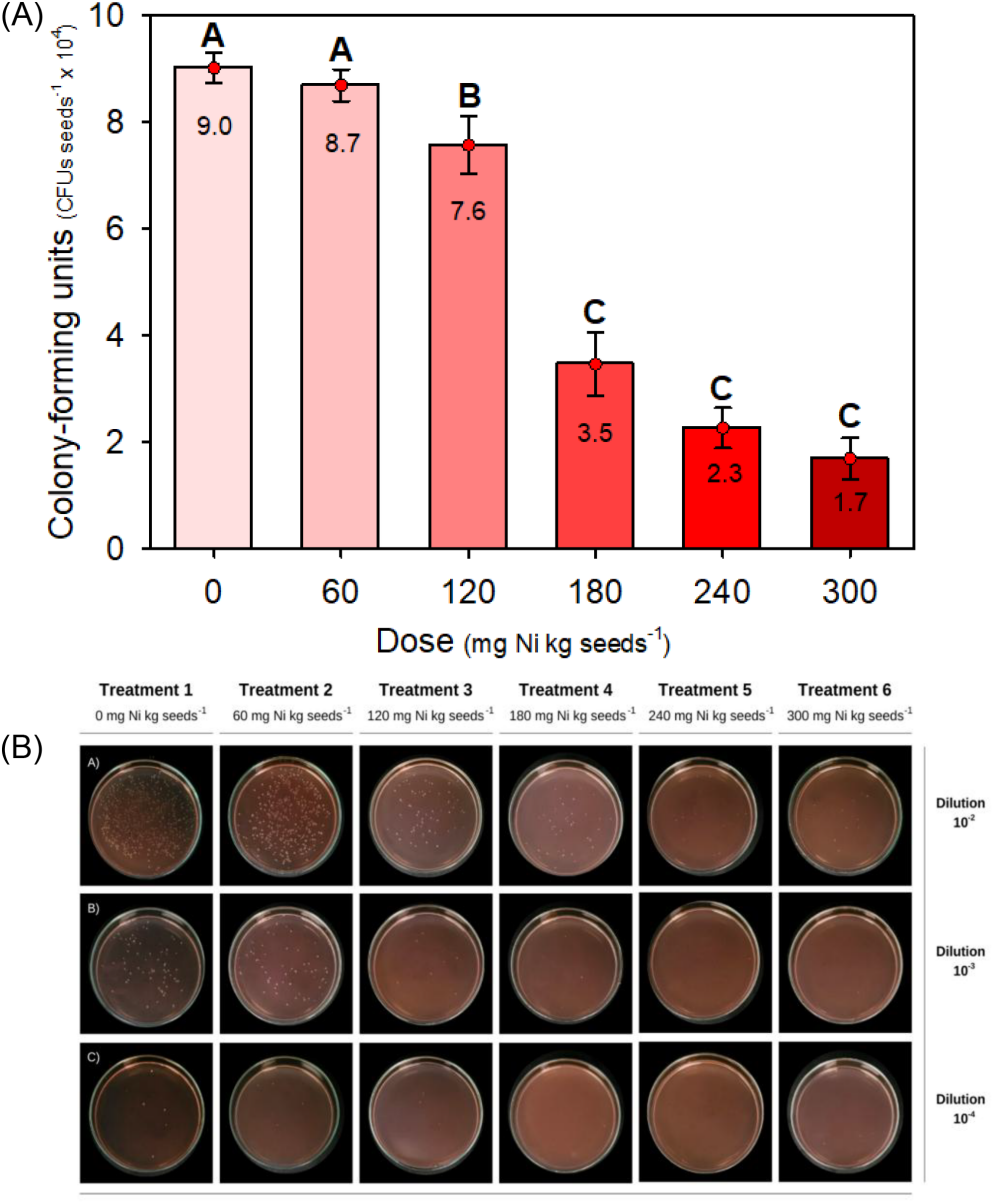
**(A)** Colony-forming units (CFUs) recovered from soybean seeds seven days after treatment with Ni and inoculation with *Bradyrhizobium* spp. Embrapa Soja, Londrina, PR. Values are means of *n* = 4. Bars with different letters are significantly different by the LSD test at *p* < 0.10. **(B)** Colony formation by *Bradyrhizobium* spp. recovered from soybean seeds treated with different doses of Ni and inoculated with *Bradyrhizobium*. Doses of 0, 60, 120, 180, 240, and 300 mg Ni kg seed^−1^.

As shown in **Figure 3A**, Ni doses above 120 mg kg^−1^ seed had a detrimental effect on the recovery of *Bradyrhizobium*, reducing the CFUs per seed to levels significantly below the optimal threshold required for effective nodulation and plant growth. At the lowest Ni dose (60 mg kg^-1^), CFU counts remained high, approximately 8.7 × 10^4^ CFU seed^-1^, compared with 9.0 × 10^4^ CFU seed^-1^ in the control (0 mg kg^-1^), indicating minimal adverse effects on *Bradyrhizobium* viability. However, a progressive decline in CFU counts occurred as the Ni dose increased from 120 mg kg^-1^ onwards.

The dilution series in **Figure 3B** (A: 10^-2^, B: 10^-3^, and C: 10^-4^) provides a clear visual representation of how increasing Ni doses negatively influenced the microbial population on the soybean seeds. Across all dilutions (10^-2^, 10^-3^, and 10^-4^), the number of colonies was visibly higher on the plates from the control and the 60 mg kg^-1^ treatment, indicating optimal conditions for the survival and recovery of *Bradyrhizobium* cells. At the highest Ni doses (240 and 300 mg Ni kg^-1^), colonies were sparse or nearly absent, particularly in the 10^-3^ and 10^-4^ dilutions, suggesting that Ni doses above 240 mg kg^−1^ drastically reduced the viability of *Bradyrhizobium* cells. These results emphasize the importance of optimizing Ni doses in agricultural applications to maintain inoculant efficacy.

### Nitrogenase activity response to nickel dose

Since one of the goals of treating soybean seeds with Ni is to enhance BNF, we examined the effects of Ni treatment on the production of ethylene from acetylene, an indicator of nitrogenase activity, using the ARA method in pot experiments (*Experiment II).* As shown in **Figure 1**, the ethylene concentration (µL L^-1^) varied significantly as a function of both Ni dose (*p* ≤ 0.001) and sampling time in days after emergence (DAE) (*p* ≤ 0.001). However, there was no significant effect of the interaction between Ni dose and sampling time (*p* ≤ 0.797), suggesting that the response to Ni fertilization was consistent across phenological stages.

At all sampling times, ethylene levels were highest in the control and the 60 mg Ni kg^−1^ treatment, indicating potentially greater nitrogenase activity at these lower Ni doses. Ethylene concentrations dropped significantly at a dose of 120 mg Ni kg^-1^ and were lowest in the 180, 240, and 300 mg Ni kg^-1^ treatments, suggesting a dose-dependent reduction in ethylene concentrations.

Regardless of Ni dose, ethylene concentrations were lowest at 36 DAE and increased gradually until peaking around 54 DAE. Ethylene levels at 60 DAE were comparable to those at 54 DAE, indicating a stabilization or plateau in ARA activity in the later stages of development. These results indicate that treating soybean seeds with lower doses of Ni may promote nitrogenase activity during the early flowering stage, likely enhancing BNF and increasing ARA. Conversely, higher Ni doses appear to inhibit ethylene production, suggesting a negative impact on nitrogenase activity.

### Nickel effects on soil CO_2_ flux and microbial activity

To evaluate the impact of seed treatment with Ni on microbial activity and root respiration, an Arduino prototype (**Figure 2**) was used to assess soil concentrations of CO_2_ in pot experiments (*Experiment III*). The results, which are presented in **Figure 2**, demonstrated that the CO_2_ concentration (µL L^-1^) in soil cultivated with soybean was significantly affected by both Ni dose (*p* ≤ 0.001) and sampling time (DAE) (*p* ≤ 0.001). However, the effect of the interaction between Ni dose and sampling time was not significant (*p* = 0.823), suggesting that the response to Ni dose was consistent across phenological stages.

CO_2_ levels were consistently higher in the control treatment and the 0 and 60 mg Ni kg^−1^ seed treatments across most of the soybean growth cycle and peaked around the flowering stage (R_1_ to R_4_ stages). This trend suggests a direct correlation of these specific Ni doses with enhanced microbial and root respiration activity, consistent with increased CO_2_ levels in the soil environment. The CO_2_ concentrations gradually decreased after the flowering stage across all treatments, indicating reductions in microbial activity and root respiration as the plants progressed to later growth stages.

Treatments with higher doses of Ni (120, 180, 240, and 300 mg Ni kg^−1^) consistently exhibited lower CO_2_ levels than the control and lower Ni dose treatments, suggesting that treating seeds with elevated Ni doses inhibited microbial processes and reduced root activity. These effects are also consistent with the decreases in *Bradyrhizobium* recovery (**Figure 3**).

### Field performance and yield response to nickel seed treatment

This study was conducted across three field sites characterized by a natural gradient of soil-available Ni (0.4–0.6 mg dm^-3^) and pronounced textural variability, ranging from sandy to clayey soils. Importantly, despite this edaphic variability, the response pattern to seed-applied Ni was consistent across environments, with similar dose–response behavior observed at all sites.

To determine whether the effects of Ni seed treatment on microbial activity in pot experiments translated into changes in soybean productivity, field experiments were performed over two growing seasons (2022/23 and 2023/24) at three sites in Brazil: Selvíria, Botucatu, and Sengés (*Experiment IV*). The results are presented in **Table 1**.

**Table 1:** Number of nodules (NN); nodule dry weight (NDW), shoot dry weight (SDW) and root dry weight (RDW) per plant; 100-grain weight (100GW); and soybean grain yield (GY) as a function of nickel (Ni) seed treatment dose.

Treatment mg Ni kg^-1^	Sengés – PR											
	NN		NDW		SDW		RDW		100GW		GY	
	n°		g plant^−1^						g		kg ha^−1^	
	2022/23	2023/24	2022/23	2023/24	2022/23	2023/24	2022/23	2023/24	2022/23	2023/24	2022/23	2023/24
0	60.0 b	55.1 bc	226 c	220 bc	16.0 abc	16.8	4.0	3.8	17.0 bc	18.0	4251	4307 bc
60	64.2 ab	61.0 a	242 b	244 a	16.3 ab	17.0	4.1	3.8	17.2 ab	18.1	4416	4439 ab
120	68.0 a	60.1 ab	256 a	240 ab	16.4 a	17.8	4.1	3.9	17.6 a	18.1	4324	4566 a
180	60.0 b	53.2 c	226 c	213 c	15.8 abc	16.9	4.0	3.7	16.8 bc	18.0	4272	4396 ab
240	51.2 c	45.5 d	192 d	182 d	15.8 bc	16.5	4.0	3.8	16.6 c	17.9	4205	4280 bc
300	48.4 c	43.7 d	182 d	175 d	15.6 c	16.2	3.9	3.7	16.5 c	17.9	4177	4078 c
Test F												
*p*-value	0.001	0.001	0.001	0.001	0.203	0.398	0.208	0.918	0.0240	0.902	0.209	0.043
C.V. (%)	6.75	7.83	4.49	7.95	3.09	6.36	3.06	7.80	2.48	2.22	4.99	4.37
Botucatu - SP												
0	78.6 bcd	49.8 a	304 b	199 a	21.1	15.0 ab	5.0 bc	3.6	19.7	17.6	4932 bc	4175 b
60	84.1 ab	53.3 a	330 a	213 a	21.5	15.7 a	5.4 a	3.8	20.2	17.7	5048 ab	4494 a
120	89.5 a	54.0 a	346 a	215 a	21.6	14.0 bc	5.4 a	3.6	20.5	17.7	5100 a	4253 b
180	79.0 bc	44.7 b	304 b	179 b	20.8	13.9 bc	5.2 ab	3.5	19.5	17.7	4959 bc	4240 b
240	77.1 cd	40.9 bc	308 b	163 bc	20.7	13.3 c	5.2 ab	3.5	19.3	17.2	4931 bc	3952 c
300	73.1 d	38.2 c	301 b	153 c	20.6	13.6 c	4.9 c	3.5	19.2	17.2	4847 c	3917 c
Test F												
*p*-value	0.003	0.001	0.002	0.001	0.553	0.019	0.057	0.810	0.268	0.565	0.036	0.001
C.V. (%)	5.87	7.24	4.51	7.19	4.41	6.63	4.51	8.77	4.25	2.82	5.1	3.65
Selvíria - MS												
0	63.6 b	52.5 b	249 b	210 b	19.0	15.9	4.7 bc	3.7	19.1	17.8	5566 b	4241 c
60	68.2 b	57.2 a	267 b	229 a	19.3	16.3	4.9 b	3.8	19.4	17.9	5772 a	4466 a
120	74.9 a	57.0 a	293 a	228 a	19.4	15.9	5.3 a	3.8	19.3	17.9	5756 a	4410 ab
180	66.3 b	48.9 b	259 b	196 b	18.7	15.4	4.7 bc	3.6	18.9	17.9	5590 b	4318 bc
240	56.4 c	43.2 c	221 c	172 c	18.7	14.9	4.6 bc	3.7	18.7	17.6	5466 b	4116 d
300	56.4 c	40.9 c	221 c	164 c	18.5	14.9	4.5 c	3.6	18. 5	17.6	5509 b	3998 e
Test F												
*p*-value	0.001	0.001	0.001	0.001	0.652	0.144	0.042	0.777	0.290	0.370	0.012	0.001
C.V. (%)	6.67	6.46	6.53	6.47	4.70	5.57	6.34	5.94	3.22	1.70	6.16	2.22

*Data were analyzed by analysis of variance (ANOVA), and when significant differences were identified, means were compared using Fisher’s protected least significant difference (LSD) test at p < 0.10. Means followed by different letters differ from each other within the same column, for the 2022/23 and 2023/24 growing seasons.

Treating soybean seeds with Ni significantly affected both the number of nodules (NN) and the nodule dry weight (NDW) at all sites in both growing seasons (**Table 1**). Among the individual treatments, both the 60 and 120 mg Ni kg^-1^ treatments significantly increased NN and NDW compared with the control (*p* < 0.01). In the 120 mg Ni kg^-1^ treatment, NN and NDW both increased by 17.8% (2022/23) and 8.6% (2023/24) in Selvíria, 13.9% in Botucatu (2022/23), and 13.3% in Sengés (2022/23). At doses ≥ 180 mg Ni kg^-1^, NN and NDW dropped sharply by 20%–30%, indicating detrimental effects on *Bradyrhizobium* spp. survival or root colonization.

Although shoot dry weight (SDW) and root dry weight (RDW) did not differ significantly among the treatments, treating soybean seeds with Ni did significantly affect soybean grain yield and associated yield components (**Table 1**). Grain yields were consistently highest in the 120 mg Ni kg^-1^ treatment at all locations, with statistically significant increases over the control. In Selvíria, the grain yield in the 120 mg Ni kg^−1^ treatment reached 5756 kg ha^-1^ in 2022/23 and 4410 kg ha^-1^ in 2023/24, representing increases of 3.4% and 4.0%, respectively, compared to the control. Similarly, in Botucatu, yields peaked at 5100 kg ha^-1^ and 4253 kg ha^-1^ in the 120 mg Ni kg^-1^ treatment in the two seasons, corresponding to improvements of 3.4% and 1.9% over the control. In Sengés, the same dose resulted in yields of 4324 kg ha^-1^ and 4566 kg ha^-1^ in 2022/23 and 2023/24, corresponding to increases of 1.7% and 6.0% compared to the control. The 100-grain weight followed a similar trend. The 120 mg Ni kg^-1^ treatment increased the 100-grain weight by up to 3.5%, while higher doses resulted in reductions of 2.5%–5.9%. Shoot and root dry weights were also maximized at 120 mg Ni kg^-1^, with average increases of 5%–7% over the control, although the differences were not always statistically significant.

At higher Ni rates (240 and 300 mg kg^-1^), a clear yield penalty was observed. In Selvíria, yields dropped by 1.0%–4.4% in the 240 and 300 mg Ni kg^-1^ treatments compared to the control, while in Botucatu, the 300 mg Ni kg^-1^ treatment reduced grain yield by 2.1%–6.2%. In Sengés, the 300 mg Ni kg^-1^ treatment reduced grain yield by 1.7% in 2022/23 and by 5.3% in 2023/24 compared to the control, confirming that excessive Ni levels.

### Leaf nutrient composition response to nickel seed treatment

To explore the mechanisms underlying the effects of Ni seed treatment on soybean productivity, the levels of macro- and micronutrients in diagnostic leaves of soybean in the field experiments were determined at the R_2_ phenological stage (full flowering) (*Experiment IV*). As shown in **Supplementary Table 3**, leaf macronutrient concentrations did not differ significantly among the Ni treatments in all site-years. The levels of N, P, K, Ca, Mg, and S were stable, indicating that Ni application did not impair nutrient uptake or translocation. Similarly, micronutrient concentrations (**Supplementary Table 4**) showed no significant changes, although a slight numerical increase in leaf Ni content was observed, particularly at higher Ni doses. Across all sites, leaf Ni content increased by 10%–20% in the 300 mg Ni kg^-1^ treatment compared to the control (which had values ranging from 0.42 to 0.60 mg kg^-1^), although this increase was not statistically significant.

### Enhancement of photosynthetic performance by optimal nickel doses

The effects of Ni application on photosynthetic parameters were examined by analyzing pigment accumulation and RuBisCO activity in leaves and gas exchange variables at the R_2_ stage (**Tables 2**–**4**). As shown in **Table 2**, the 120 mg Ni kg^−1^ treatment increased total chlorophyll (Chl t) by up to 25.2% in Botucatu (2022/23) and 8.4% in Selvíria (2023/24) compared with the control treatment. At the highest Ni dose, chlorophyll levels decreased by 7.5%–10% compared to the 120 mg Ni kg^-1^ treatment in Botucatu. Consistent with the increase in chlorophyll content, the 120 mg Ni kg^-1^ treatment significantly enhanced the net photosynthetic rate (*A*) across all locations (**Table 3**). In Sengés, *A* increased from 15.0 µmol CO_2_ m^-2^ s^-1^ in the control to 17.7 µmol CO_2_ m^-2^ s^-1^ in the 120 mg Ni kg^-1^ treatment (+18%) in 2022/23 and from 12.3 to 13.4 µmol CO_2_ m^-2^ s^-1^ (+8.9%) in the corresponding treatments in 2023/24. Carboxylation efficiency (CE) also improved, peaking at 0.065–0.071, representing an average increase of 23% over the control. RuBisCO activity also peaked at intermediate doses (60–120 mg Ni kg^-1^) (**Table 4**). These results indicate that the increases in productivity at lower Ni doses (≤ 120 mg Ni kg^-1^) reflect increases in photosynthetic parameters.

**Table 2:** Concentrations of photosynthetic pigments (chlorophyll *a* – Chl *a*; chlorophyll *b* – Chl *b*; total chlorophyll – Chl t; and total carotenoids – Car t) in diagnostic soybean leaves at the R_2_ phenological stage as a function of nickel (Ni) seed treatment dose.

Treatment mg Ni kg^-1^	Sengés - PR							
	Chl *a*		Chl *b*		Chl t		Car t	
	mg g^-1^ fresh weight							
	2022/23	2023/24	2022/23	2023/24	2022/23	2023/24	2022/23	2023/24
0	929	1073 bc	407	459	1336	1532 a	445	451
60	1005	1123 ab	415	459	1420	1531 a	486	452
120	988	1145 a	427	452	1415	1598 a	442	449
180	974	1073 bc	455	448	1429	1571 a	411	456
240	971	1057 cd	485	478	1455	1535 a	478	441
300	964	998 d	478	447	1443	1445 b	468	562
Test F								
*p*-value	0.686	0.015	0.140	0.674	0.504	0.043	0.176	0.876
C.V. (%)	6.71	4.7	10.54	6.2	6.24	3.9	9.11	5.3
Botucatu - SP								
0	1000 c	1030 ab	500 bc	485	1500 c	1515	500	428
60	1474 a	1064 a	598 a	486	2072 a	1550	495	447
120	1363 b	1022 abc	525 b	482	1889 b	1505	499	447
180	1004 c	977 cd	453 cd	490	1457 cd	1467	518	464
240	972 c	996 bcd	440 cd	474	1411 d	1470	508	448
300	993 c	962 d	393 d	497	1386 d	1459	517	443
Test F								
*p*-value	0.001	0.028	0.002	0.876	0.001	0.200	0.866	0.839
C.V. (%)	5.26	4.1	11.48	5.3	3.98	3.66	6.38	8.2
Selvíria – MS								
0	1204 c	1051 bc	460	472	1663 cd	1523 ab	433	440
60	1340 a	1069 ab	480	472	1819 a	1541 a	463	450
120	1283 ab	1083 a	477	468	1760 ab	1551 a	444	448
180	1229 bc	1050 bc	473	469	1702 bc	1519 ab	468	460
240	1195 c	1026 c	482	476	1677 bcd	1503 b	466	445
300	1118 d	980 d	476	472	1593 d	1452 c	465	452
Test F								
*p*-value	0.003	0.001	0.929	0.978	0.006	0.006	0.206	0.846
C.V. (%)	5.01	2.5	6.60	3.2	4.04	2.0	5.00	4.9

*Data were analyzed by analysis of variance (ANOVA), and when significant differences were identified, means were compared using Fisher’s protected least significant difference (LSD) test at p < 0.10. Means followed by different letters differ from each other within the same column, for the 2022/23 and 2023/24 growing seasons.

**Table 3:** Net photosynthetic rate (*A* − µmol CO_2_ m^-2^ s^-1^), stomatal conductance (*gs* − mol H_2_O m^-2^ s^-1^), intercellular CO_2_ concentration (*Ci* − µmol CO_2_ mol^-1^), leaf transpiration rate (*E* − mmol H_2_O m^-2^ s^-1^), water use efficiency (WUE − μmol CO_2_ (mmol H_2_O)^−1^), and carboxylation efficiency (CE − dimensionless) in diagnostic soybean leaves at the R_2_ phenological stage as a function of nickel (Ni) seed treatment dose.

Treatment mg Ni kg^-1^	Sengés - PR											
	*A*		*gs*		*Ci*		*E*		WUE		CE	
	2022/23	2023/24	2022/23	2023/24	2022/23	2023/24	2022/23	2023/24	2022/23	2023/24	2022/23	2023/24
0	15.0 c	12.3 ab	0.23	0.19	284	198	4.6	3.2	3.3 c	3.9	0.053 c	0.062 bc
60	16.1 b	12.7 ab	0.25	0.19	278	200	4.5	3.2	3.6 ab	4.0	0.058 b	0.063 b
120	17.7 a	13.4 a	0.25	0.20	273	192	4.6	3.1	3.9 a	4.3	0.065 a	0.070 a
180	15.7 bc	11.9 bc	0.23	0.19	275	194	4.5	3.2	3.5 bc	3.8	0.057 bc	0.061 bc
240	14.8 c	12.1 bc	0.23	0.20	278	193	4.3	3.2	3.5 bc	3.8	0.053 c	0.063 b
300	15.1 bc	11.0 c	0.20	0.21	285	194	4.3	3.1	3.6 bc	3.5	0.053 c	0.057 c
Test F												
*p*-value	0.002	0.048	0.592	0.807	0.417	0.789	0.503	0.995	0.097	0.239	0.002	0.025
C.V. (%)	5.51	7.59	18.8	9.11	3.34	4.57	7.21	6.87	7.36	10.2	6.42	6.96
Botucatu - SP												
0	12.2 c	13.4 b	0.180 cd	0.22	235	235	3.5	3.1	3.5	4.3	0.052 d	0.06
60	13.1 b	13.4 b	0.203 b	0.20	232	211	3.7	3.1	3.6	4.3	0.057 bc	0.06
120	14.0 a	14.4 a	0.223 a	0.20	222	207	3.7	3.1	3.8	4.6	0.063 a	0.07
180	13.0 b	13.2 bc	0.190 c	0.20	223	190	3.7	3.1	3.5	4.3	0.058 b	0.07
240	12.1 c	12.7 bc	0.170 de	0.20	222	199	3.5	3.1	3.5	4.2	0.054 cd	0.06
300	12.3 c	12.4 c	0.168 e	0.21	226	197	3.5	3.1	3.6	4.0	0.054 cd	0.06
Test F												
*p*-value	0.002	0.008	0.001	0.629	0.103	0.258	0.561	0.998	0.481	0.309	0.001	0.357
C.V. (%)	4.34	4.96	4.96	9.07	3.25	12.73	7.1	6.73	5.82	8.38	5.03	13.5
Selvíria - MS												
0	11.4 c	12.9 b	0.165 cd	0.21	212 a	217	3.2	3.2	3.5 b	4.1 b	0.053 c	0.06
60	11.8 bc	13.0 b	0.185 ab	0.20	207 a	206	3.1	3.2	3.8 ab	4.2 ab	0.058 bc	0.06
120	12.5 a	13.9 a	0.192 a	0.20	192 b	200	3.1	3.1	4.1 a	4.5 a	0.068 a	0.07
180	12.3 a	12.5 b	0.175 bc	0.20	189 b	192	3.1	3.1	4.1 a	4.0 b	0.065 a	0.07
240	11.9 b	12.4 bc	0.158 d	0.20	187 b	196	3.1	3.1	4.0 a	4.0 b	0.062 ab	0.06
300	11.0 d	11.7 c	0.145 e	0.21	188 b	195	3.1	3.1	3.7 ab	3.8 b	0.058 bc	0.06
Test F												
*p*-value	0.001	0.059	0.001	0.631	0.007	0.110	0.972	0.984	0.095	0.086	0.009	0.116
C.V. (%)	2.28	5.10	5.08	5.73	4.87	6.01	6.11	4.01	8.23	7.11	8.50	8.62

*Data were analyzed by analysis of variance (ANOVA), and when significant differences were identified, means were compared using Fisher’s protected least significant difference (LSD) test at p < 0.10. Means followed by different letters differ from each other within the same column, for the 2022/23 and 2023/24 growing seasons.

**Table 4:** N-Ureides (µmol N g^-1^ DW) and activities of urease (µmol NH_4_^+^ g^-1^ DW h^-1^), nitrate reductase (µmol NO_2_^-^ g^-1^ DW h^-1^), and RuBisCO (µmol CO_2_ g^-1^ DW h^-1^) in diagnostic soybean leaves at the R_2_ phenological stage as a function of nickel (Ni) seed treatment dose.

Treatment mg Ni kg^-1^	Sengés - PR							
	N-Ureides		Urease		Nitrate reductase		RuBisCO	
	2022/23	2023/24	2022/23	2023/24	2022/23	2023/24	2022/23	2023/24
0	19.1 c	19.3 ab	5.8 b	4.6 b	151	144	4.6 abc	3.9
60	20.4 b	19.9 ab	5.7 b	4.7 b	153	145	5.1 a	4.0
120	22.6 a	21.1 a	5.5 b	4.5 b	157	140	5.0 ab	4.4
180	19.1 c	18.7 bc	5.9 b	5.6 ab	153	141	4.6 bc	4.6
240	17.6 d	19.1 bc	7.4 a	5.8 a	152	140	4.3 c	4.1
300	17.6 d	17.4 c	7.7 a	6.3 a	147	141	4.2 c	4.1
Test F								
*p*-value	0.001	0.052	0.001	0.037	0.193	0.781	0.0341	0.666
C.V. (%)	4.94	7.71	8.56	15.7	3.33	4.46	8.93	15.87
Botucatu - SP								
0	11.2 bc	19.8 b	3.5 c	4.2 b	157 a	188	3.3 bc	3.1 b
60	13.8 a	19.7 b	3.4 c	4.2 b	158 a	168	4.5 a	3.4 b
120	13.3 a	21.3 a	3.3 c	4.7 ab	156 a	166	4.8 a	3.9 a
180	11.8 b	19.3 bc	4.3 b	4.8 ab	146 b	152	3.7 b	3.1 b
240	11.0 bc	18.8 bc	5.0 a	5.4 a	144 b	159	3.6 bc	3.1 b
300	10.4 c	18.3 c	5.0 a	5.4 a	136 c	157	3.2 c	3.1 b
Test F								
*p*-value	0.012	0.007	0.001	0.058	0.001	0.258	0.002	0.049
C.V. (%)	8.24	4.78	13.5	13.7	4.0	12.73	10.7	11.5
Selvíria - MS								
0	15.4 c	19.6 b	4.6 b	4.4 c	185 bc	166	4.4 abc	3.5 b
60	17.2 b	19.8 b	4.6 b	4.4 c	201 a	157	5.0 a	3.7 b
120	18.5 a	21.2 a	4.4 b	4.6 bc	194 ab	153	4.9 ab	4.2 a
180	14.9 cd	19.0 bc	4.8 b	5.2 ab	180 c	147	4.5 bc	3.8 ab
240	14.2 de	18.9 bc	6.1 a	5.6 a	179 c	150	4.2 c	3.6 b
300	13.7 e	17.8 c	6.5 a	5.8 a	179 c	149	4.1 c	3.6 b
Test F								
*p*-value	0.001	0.007	0.001	0.012	0.018	0.126	0.035	0.095
C.V. (%)	5.65	5.19	9.11	11.8	5.0	6.31	7.05	8.34

*Data were analyzed by analysis of variance (ANOVA), and when significant differences were identified, means were compared using Fisher’s protected least significant difference (LSD) test at p < 0.10. Means followed by different letters differ from each other within the same column, for the 2022/23 and 2023/24 growing seasons.

### Influence of nickel seed treatment on enzymatic nitrogen metabolism

To evaluate the effects of Ni seed treatment on N metabolism, the leaf concentration of N-ureides, a proxy for BNF, and leaf activities of urease and NR were determined in soybean plants in the field experiment at the R_2_ stage (**Table 4**, *Experiment IV*). Compared with the control, the 120 mg Ni kg^-1^ treatment increased the concentration of N-ureides by up to 18%. Conversely, N-ureide concentrations declined by 8%–13% in the 240 and 300 mg Ni kg^-1^ treatments. Similarly, NR activity peaked at intermediate doses. As expected, urease activity increased linearly with Ni dose. In Sengés, urease activity was 32% higher in the 300 mg Ni kg^-1^ treatment than in the control. However, the increase in urease activity was not accompanied by improved BNF or yield.

### Optimal nickel dose for soybean seed treatment

**Figure 4:**
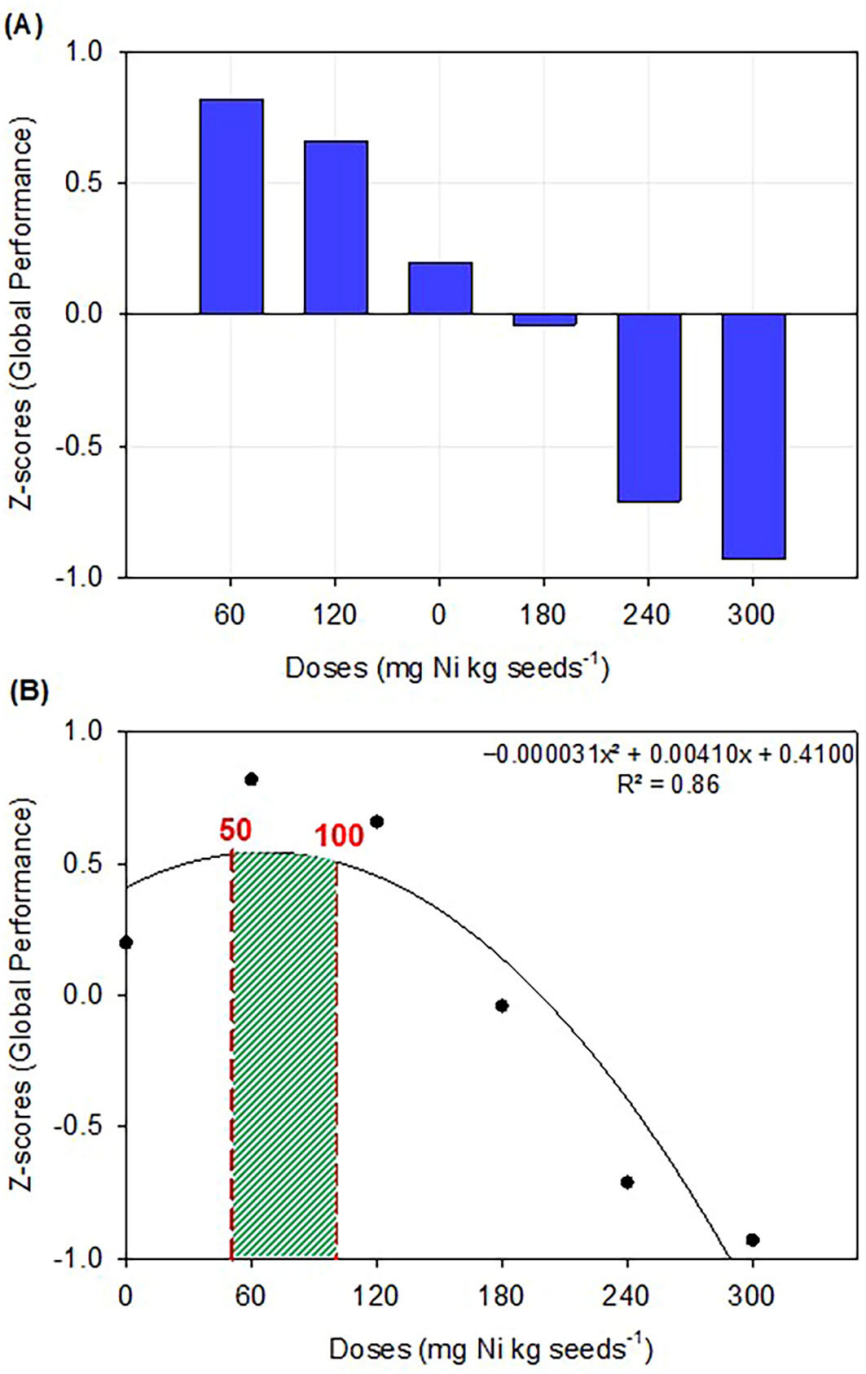
**(A)** Mean Z-score per treatment. The bars represent the average standardized (Z-scored) value across agronomic variables included in the analysis. **(B)** Multiple polynomial regression modeling of the mean Z-score as a function of Ni dose applied in seed treatment. Prior to analysis, all response variables were standardized (Z-score transformation) to allow for comparison across different scales and units. The mean Z-score was then calculated for each treatment and used as the dependent variable in the regression model.

Z-score transformation enabled the aggregation of heterogeneous variables on a common scale, enhancing the multivariate interpretability of treatment effects (**Figure 4**). Applying a second-order polynomial regression model to the mean standardized Z-scores revealed a statistically significant quadratic response (*p* < 0.05) the integrated agronomic traits. The model identified an optimal dose of 66.2 mg Ni kg^-1^ seeds at the apex of the response curve (**Figure 4B**), corresponding to the maximum overall standardized performance across all evaluated variables. Beyond this critical threshold, the mean Z-scores declined consistently.

## Discussion

### Biological nitrogen fixation response to nickel seed treatment

Ni is required for the activity of microbial hydrogenases, important enzymes that reduce the energy needed for N_2_ reduction by nitrogenase (Nunes et al., 2003; Bagyinka, 2014). Consequently, providing Ni via seed treatment could enable early biosynthesis and activation of hydrogenase, increasing BNF efficiency. Hydrogenase activity can be assessed by ARA measurement, which is an important tool for evaluating BNF throughout the growth cycle due to its simplicity, low cost and high sensitivity (Devi and Sinclair, 2013).

Consistent with the results of previous studies showing that Ni levels influence ARA activity (Ureta et al., 2005; Yusuf et al., 2011; Lavres et al., 2016; Freitas et al., 2019; Rengel et al., 2022), we found that regardless of phenological stage, Ni doses of 0 and 60 mg kg^-1^ increased ethylene production (*Experiment II*). Supporting these results, Lavres et al. (2016) reported that treating soybean seeds with different doses of Ni increased BNF by an average of 10%, significantly contributing to N supply, especially in grains. In another assessment of the effects of different Ni doses on soybean, Freitas et al. (2019) observed that doses of up to 60 mg Ni kg seeds^-1^ increased nitrogenase activity, enhancing NN, NDW, photosynthesis, and grain yield. However, the same study showed that Ni doses above 125 mg Ni kg seeds^-1^ reduced these parameters and were associated with symptoms of Ni toxicity. Similar effects were observed in our study at Ni doses of 120, 180, 240, and 300 mg kg^-1^.

High CO_2_ concentrations in the environment enhance plant growth, increase symbiotic activity, and promote nodulation in soybean inoculated with *Bradyrhizobium japonicum* (Akley et al., 2022). According to Dacal et al. (2019), gas emission processes and soil quality are influenced by microbial activity, root respiration, decomposition of plant residues, and oxidation of organic matter. The soil bacterial community engages in intense decomposition activity, leading to the oxidation of organic compounds and the emission of CO_2_ into the gas phase (Atere et al., 2020). Thus, higher levels of emission of CO_2_ are indicative of soils with high respiration rates, microbial activity, and microbial diversity (Sun et al., 2024). The use of an Arduino prototype to evaluate CO_2_ release (Kormann et al., 2024) in *Experiment III* allowed a comprehensive and continuous understanding of gas dynamics throughout the soybean crop cycle.

Throughout most of the experimental period, CO_2_ concentrations were higher in the control treatment and in the treatments with Ni doses of 60 and 120 mg kg^-1^; these treatments also led to the highest recovery of *Bradyrhizobium* from treated seeds. In all treatments, the CO_2_ concentration increased over time until the flowering stage of soybean and declined thereafter. Increases in CO_2_ concentrations may indicate greater microbial activity in the soil (Zapata et al., 2021). Larger microbial populations suggest an increase in microbial biomass and, consequently, greater C immobilization, which is important from the perspective of C sequestration (Rao et al., 2021).

### Nickel toxicity and *Bradyrhizobium*–soybean symbiosis

#### Nickel toxicity and symbiosis establishment between soybean and *Bradyrhizobium*

Adequate inoculation of soybean seeds ensures the presence of diazotrophic bacteria in the necessary quantity and quality for early symbiosis establishment (Moretti et al., 2020a; Martins et al., 2022). The bacterial strains recommended for use in commercial inoculants for soybean cultivation in Brazil belong to the species *Bradyrhizobium japonicum* (SEMIA 5079 = CPAC 15), *B. diazoefficiens* (SEMIA 5080 = CPAC 7), and *B. elkanii* (SEMIA 5019 and SEMIA 587) (Zilli et al., 2021; Bender et al., 2022). These strains have been demonstrated to be highly efficient in BNF (Hungria and Vargas, 2000), providing adequate N to support high soybean yields while maintaining low production costs. In Brazil and other countries, quality control of commercial inoculated microorganisms is based on cell concentration and the absence of contaminants (Santos et al., 2020; Moretti et al., 2021). The industry has made significant progress in developing products containing high cell concentrations, and concentration control ensures that farmers receive high-quality products (Santos et al., 2020).

However, the number of viable cells on inoculated seeds at the time of sowing is even more important than the concentration of the inoculant, as it influences the speed of nodulation and the occupancy of the inoculated bacteria (Hungria et al., 2017). In established areas of soybean cultivation, a population of nodulating rhizobia is already present in the soil, and the results of field experiments have indicated that the inoculant should provide, in theoretical estimates, at least 1.2×10^6^ cells per seed, ensuring that at least 7%–8% (8×10^4^ to 1×10^5^ viable cells) are recovered at the time of sowing (Hungria et al., 2017).

The adverse effects of treating seeds with chemical products on bacterial survival (Akley et al., 2022; Pinto et al., 2023) can be mitigated by the use of cell-protecting additives in the inoculant formulation (Santos et al., 2020) or other methods of delivery, such as in-furrow inoculation, that physically separate the additive and the bacteria (Hungria et al., 2010). Even though the inoculant used in the present study had a high cell concentration of at least 5×10^9^ CFU mL^-1^ and contained protectants, the number of cells recovered from seeds treated with a Ni dose of 180 mg kg^-1^ was approximately one-third to one-fourth of the ideal minimum value for promoting rapid nodulation and high nodular occupancy by inoculant strains, demonstrating that doses higher than 120 mg Ni kg^-1^ compromise the survival of *Bradyrhizobium.*

In other studies, conducted in our laboratory, a reduction of up to 98% of *Bradyrhizobium* cells was observed in seeds treated with incompatible inputs. In the field, incompatibility between the inoculant and the chemical treatment decreased soybean nodulation by 14% in soils with an established *Bradyrhizobium* population and by 70% in soils without an established population (Pinto et al., 2023). Similar results were reported for the application of pesticides as seed treatments (Sartori et al., 2023). Our observations underscore the importance of precise Ni management for *Bradyrhizobium* survival.

### Nickel effects on nodulation and plant establishment

Ni doses above a threshold value adversely affect nodulation and overall plant health (Lavres et al., 2016). The architecture of root development—including root hair formation, primary root elongation, and lateral root initiation—represents a complex interaction between root growth dynamics and nutrient availability (Moretti et al., 2020b). Higher Ni concentrations impair nodule development and function, which are essential for BNF and plant growth (Freitas et al., 2018). Ni application rates up to 60 mg kg^−1^ consistently enhanced nodulation, and physiological vigor without adversely affecting 100-grain weight, supporting the hypothesis that moderate doses of seed-applied Ni are agronomically safe and effective. By contrast, higher doses of 180–300 mg kg^−1^ led to sharp declines in NN and NDW, as well as N metabolism associated with BNF. These findings align with the results of previous studies indicating that Ni toxicity negatively impacts N fixation, root morphology, and phenylpropanoid pathway activity, ultimately compromising plant establishment and yield. The influence of Ni on the regulation of nodulation-related gene expression appears to be mainly mediated by its effects on phenylalanine ammonia-lyase (PAL) activity and *nod* gene expression. The expression of PAL, a key enzyme in the phenylpropanoid pathway, is upregulated in response to metal stress, leading to increased synthesis of phenols, flavanones, and isoflavanones (Shen et al., 2022).

### Nickel effects on photosynthesis and productivity

The decrease in the photosynthetic rate (*A*) at higher Ni doses, combined with elevated intercellular CO_2_ concentrations (*Ci*)—partly due to reduced stomatal conductance (*gs*), i.e., stomatal closure—suggests that decreased nodulation not only has direct detrimental effects on photosynthesis but also compromises the carbon fixation capacity of soybean plants (Pietrini et al., 2015).

Importantly, our findings indicate that an adequate supply of Ni positively regulates stomatal opening and closure, directly enhancing gas exchange processes and contributing to increased biomass accumulation. The observed improvement in photosynthetic activity may have resulted from enhanced nutrient uptake and/or reduced Ni absorption. Specifically, lower Ni doses increased RDW compared to the control. A more developed root system improves soil exploration, thereby promoting greater root distribution and activity in soybean plants. This, in turn, can indirectly support key metabolic functions and facilitate successful plant establishment.

Plants that displayed more pronounced symptoms of Ni toxicity exhibited elevated urease activity. Under abiotic stress conditions such as Ni toxicity, the activities of arginine decarboxylase and ornithine decarboxylase are typically upregulated. These enzymes catalyze arginine degradation, releasing spermidine and spermine in mitochondria and urea in the cytosol (Reis et al., 2017). Thus, the observed increase in urease activity in soybean plants exposed to Ni doses exceeding 180 mg kg^-1^ likely represents a physiological response to enhanced arginine catabolism and the consequent rise in cytosolic urea levels (Macedo et al., 2016). Furthermore, urea and ammonia can be direct products of ureide degradation through the urease pathway (Todd et al., 2006).

## Conclusions

This study demonstrates that treating soybean seeds with Ni may improve BNF and plant growth. However, precise management of Ni fertilization is required for optimal crop productivity. Higher doses, however, reduced *Bradyrhizobium* viability on seeds, nodulation and soybean yield. Therefore, an optimal Ni range of 50–100 mg Ni kg^−1^ seed is recommended to balance the benefits of BNF while avoiding toxicity. This range promotes sustainable soybean production by enhancing nitrogenase activity and nodulation without inducing toxic effects, thereby improving both nodule biomass and grain yield. This dosage represents a scientifically supported and low-risk recommendation for enhancing nitrogen fixation and soybean yield under Ni-deficient soil conditions.

## Data Availability

The datasets presented in this study can be found in online repositories. The names of the repository/repositories and accession number(s) can be found in the article/**Supplementary Material**.

## Supplementary material

Supplementary Table 1 Experimental information at the three study locations. Cropping seasons 2022/23 and 2023/24.

Supplementary Table 2 Initial chemical, physical, and biological soil properties at a depth of 0-20 cm and edaphoclimatic conditions at the three study locations.

Supplementary Table 3 Macronutrient concentrations (nitrogen – N; phosphorus – P; potassium – K; calcium – Ca; magnesium – Mg; and sulfur – S) in diagnostic soybean leaves plus petioles at the R2 phenological stage as a function of nickel (Ni) seed treatment dose. 2022/23 and 2023/24 growing seasons.

Supplementary Table 4 Micronutrient concentrations (iron – Fe; copper – Cu; zinc – Zn; manganese – Mn; boron – B; and nickel – Ni) in diagnostic soybean leaves plus petioles at the R2 phenological stage as a function of nickel (Ni) seed treatment dose. 2022/23 and 2023/24 growing seasons.

Supplementary Figure 1 Average biweekly temperatures and precipitation (mm) during the first and second growing seasons in Sengés, Botucatu, and Selvíria. Cropping seasons 2022/23 and 2023/24.

## References

[B1] Akley E. K. Rice C. W. Adotey N. Ampim P. A. Y. Vara Prasad P. V. Owusu Danquah E. . (2022). Residual Bradyrhizobium inoculation effects on soybean performance and selected soil health parameters. Agron. J. 114, 1627–1641. doi: 10.1002/agj2.21037

[B2] Alvares C. A. Stape J. L. Sentelhas P. C. Moraes Gonçalves J. L. Sparovek G. (2013). Köppen’s climate classification map for Brazil. Meteorologische. Z. 22, 711–728. doi: 10.1127/0941-2948/2013/0507

[B3] AOAC (2019). Official methods of analysis of AOAC International. 21th Edn. Ed. Latimer. Gaithersburg G. W. (Maryland: AOAC International).

[B4] Atere C. T. Osunde M. O. Olayinka A. (2020). Microbial dynamics and nutrient mineralization in soil amended with cacao pod and water hyacinth composts: implication for nitrogen fixed by soybean. Commun. Soil Sci. Plant Anal. 51, 2466–2478. doi: 10.1080/00103624.2020.1836202

[B5] Bagyinka C. (2014). How does the ([NiFe]) hydrogenase enzyme work? Int. J. Hydrogen. Energy 39, 18521–18532. doi: 10.1016/j.ijhydene.2014.07.009

[B6] Barcelos J. P. Osório d. de Souza. C. R. W. Leal A. J. F. Alves C. Z. Santos E. F. . (2017). Effects of foliar nickel (Ni) application on mineral nutrition status, urease activity and physiological quality of soybean seeds. Aust. J. Crop Sci. 11, 184–192. doi: 10.21475/ajcs.17.11.02.p240

[B7] Bender F. R. Nagamatsu S. T. Delamuta J. R. M. Ribeiro R. A. Nogueira M. A. Hungria M. (2022). Genetic variation in symbiotic islands of natural variant strains of soybean Bradyrhizobium japonicum and Bradyrhizobium diazoefficiens differing in competitiveness and in the efficiency of nitrogen fixation. Microb. Genom. 8, 000795. doi: 10.1099/mgen.0.000795, PMID: 35438622 PMC9453064

[B8] Brown P. H. Welch R. M. Cary E. E. (1987). Nickel: A micronutrient essential for higher plants. Plant Physiol. 85 (3), 801–803. doi: 10.1104/pp.85.3.801, PMID: 16665780 PMC1054342

[B9] Cantarella H. Quaggio J. Mattos D. Jr. Boaretto R. van Raij B. (2022). Bulletin 100: Fertilization and liming recommendations for the state of São Paulo. Eds. Cantarella H. Quaggio J. A. Jr. D.M. Boaretto R. M. Raij B.v. (Campinas: Agronomic Institute).

[B10] Chaintreuil C. Rigault F. Moulin L. Jaffré T. Fardoux J. Giraud E. . (2007). Nickel resistance determinants in Bradyrhizobium strains from nodules of the endemic New Caledonia legume Serianthes calycina. Appl. Environ. Microbiol. 73, 8018–8022. doi: 10.1128/AEM.01431-07, PMID: 17951443 PMC2168143

[B11] Chen C. Huang D. Liu J. (2009). Functions and toxicity of nickel in plants: Recent advances and future prospects. Clean. (Weinh) 37, 304–313. doi: 10.1002/clen.200800199

[B12] Dacal M. Bradford M. A. Plaza C. Maestre F. T. García-Palacios P. (2019). Soil microbial respiration adapts to ambient temperature in global drylands. Nat. Ecol. Evol. 3, 232–238. doi: 10.1038/s41559-018-0770-5, PMID: 30643242 PMC6420078

[B13] De Oliveira-Francesquini J. P. Hungria M. Savi D. C. Glienke C. Aluizio R. Kava V. . (2017). Differential colonization by bioprospected rhizobial bacteria associated with common bean in different cropping systems. Can. J. Microbiol. 63, 682–689. doi: 10.1139/cjm-2016-0784, PMID: 28376308

[B14] Devi M. J. Sinclair T. R. (2013). Fixation drought tolerance of the slow-wilting soybean PI 471938. Crop Sci. 53, 2072–2078. doi: 10.2135/cropsci2013.02.0095

[B15] Devi J. M. Sinclair T. R. Chen P. Carter T. E. (2014). Evaluation of elite southern maturity soybean breeding lines for drought-tolerant traits. Agron. J. 106, 1947–1954. doi: 10.2134/agronj14.0242

[B16] Devi M. J. Sinclair T. R. Vadez V. (2010). Genotypic variability among peanut (Arachis hypogea L.) in sensitivity of nitrogen fixation to soil drying. Plant Soil 330, 139–148. doi: 10.1007/s11104-009-0185-9

[B17] Donagema G. K. Viana J. H. M. Almeida B. G. Ruiz H. A. Klein V. A. Dechen S. C. F. . (2017). Soil analysis methods manual. Eds. Teixeira P. C. Donagema G. K. Fontana A. Teixeira W. G. (Brasília, DF: Embrapa Solos), 95–116.

[B18] Fabiano C. C. Tezotto T. Favarin J. Polacco J. C. Mazzafera P. (2015). Essentiality of nickel in plants: A role in plant stresses. Front. Plant Sci. 6. doi: 10.3389/fpls.2015.00754, PMID: 26442067 PMC4585283

[B19] Farooq M. Wahid A. Siddique K. H. M. (2012). Micronutrient application through seed treatments: a review. J. Soil Sci. Plant Nutr. 12, 125–142. doi: 10.4067/S0718-95162012000100011

[B20] Fehr W. R. Caviness C. E. Burmood D. T. Pennington J. S. (1977). Stage of soybean development. Special. Rep. 80, 929–931.

[B21] Freitas D. S. Rodak B. W. Carneiro M. A. C. Guilherme L. R. G. (2019). How does Ni fertilization affect a responsive soybean genotype? A dose study. Plant Soil 441, 567–586. doi: 10.1007/s11104-019-04146-2

[B22] Freitas D. S. Rodak B. W. dos Reis A. R. de Barros Reis F. de Carvalho T. S. Schulze J. . (2018). Hidden nickel deficiency? Nickel fertilization via soil improves nitrogen metabolism and grain yield in soybean genotypes. Front. Plant Sci. 9. doi: 10.3389/fpls.2018.00614, PMID: 29868070 PMC5952315

[B23] Hageman R. H. Reed A. J. (1980). Nitrate reductase from higher plants. In: San Pietro A , ed. Photosynthesis and nitrogen fixation, Methods in Enzymology, 69. (San Diego: Academic Press), 270–280. doi: 10.1016/S0076-6879(80)69026-0

[B24] Herridge D. F. Giller K. E. Jensen E. S. Peoples M. B. (2022). Quantifying country-to-global scale nitrogen fixation for grain legumes II. Coefficients, templates and estimates for soybean, groundnut and pulses. Plant Soil 474, 1–15. doi: 10.1007/s11104-021-05166-7

[B25] Hewitt E. J. Nicholas D. J. D. (1964). Enzymes of Inorganic Nitrogen Metabolism. In: Linskens H. F. Sanwal B. D. Tracey M. V. , eds. Modern Methods of Plant Analysis / Moderne Methoden der Pflanzenanalyse, 7. (Berlin, Heidelberg: Springer), 67–172. doi: 10.1007/978-3-642-48141-3_3

[B26] Hogan M. E. Swift I. E. Done J. (1983). Urease assay and ammonia release from leaf tissues. Phytochemistry 22, 663–667. doi: 10.1016/S0031-9422(00)86958-7

[B27] Hungria M. (1994). “ Metabolismo do carbono e do nitrogênio nos nódulos,” in Manual de métodos empregados em estudos de microbiologia agrícola, vol. 46 . Eds. Hungria M. Araújo R. S. ( Embrapa-SPI (EMBRAPA-CNPAF. Documentos, Brasília, DF), 247–283.

[B28] Hungria M Campo R J Souza E M Pedrosa F O (2010). Inoculation with selected strains of Azospirillum brasilense and A. lipoferum improves yields of maize and wheat in Brazil. Plant and Soil 331 (1), 413–425. doi: 10.1007/s11104-009-0262-0

[B29] Hungria M. Araujo R. S. Júnior E. B. S. Zilli J. É. (2017). Inoculum rate effects on the soybean symbiosis in new or old fields under tropical conditions. Agron. J. 109, 1106–1112. doi: 10.2134/agronj2016.11.0641

[B30] Hungria M. Mendes I. C. (2015). Nitrogen fixation with soybean: the perfect symbiosis? Ed. De Bruijn F. J. (New Jersey: Hoboken). doi: 10.1002/9781119053095.ch99

[B31] Hungria M. Vargas M. A. T. (2000). Environmental factors affecting N2 fixation in grain legumes in the tropics, with an emphasis on Brazil. Field Crops Res. 65, 151–164. doi: 10.1016/S0378-4290(99)00084-2

[B32] Kormann R. Paixão C. A. Hungria M. Nogueira M. A. Purin da Cruz S. (2024). An innovative prototype to measure carbon dioxide, ethylene, and nitric and nitrous oxides in the soil air. Commun. Soil Sci. Plant Anal. 55, 2909–2922. doi: 10.1080/00103624.2024.2378985

[B33] Kutman B. Y. Kutman U. B. Cakmak I. (2014). Effects of seed nickel reserves or externally supplied nickel on the growth, nitrogenmetabolites and nitrogen use efficiency of urea- or nitrate-fed soybean. Plant Soil 376, 261–276. doi: 10.1007/s11104-013-1983-7

[B34] Lavres J. Franco G. C. Câmara G. M. S. (2016). Soybean seed treatment with nickel improves biological nitrogen fixation and urease activity. Front. Environ. Sci. 4. doi: 10.3389/fenvs.2016.00037

[B35] Levene H. (1960). “ Robust tests for equality of variances,” in Contributions to probability and statistics: Essays in. Eds. Olkin I. Ghurye S. G. Hoeffding W. Madow W. G. Mann H. B. (Stanford, CA: Stanford University Press), 278–292. Available online at: https://books.google.nl/books?hl=en&lr=&id=ZUSsAAAAIAAJ&oi=fnd&pg=PA278&dq=levene+test+1960&ots=GchNjEvQXP&sig=twj-p6Fkiyvz_CKvrDgQNhqzvyw (Accessed Ocrober 23, 2022).

[B36] Levy C. de C. B. Mellis E. V. Murrer M. K. Inglés C. R. Daynes C. N. . (2019). Effects of nickel fertilization on soybean growth in tropical soils7. Bragantia 78, 432–443. doi: 10.1590/1678-4499.20180242

[B37] Macedo F. G. Bresolin J. D. Santos E. F. Furlan F. Silva W. T. L. Polacco J. C. . (2016). Nickel availability in soil as influenced by liming and its role in soybean nitrogen metabolism. Front. Plant Sci. 7. doi: 10.3389/fpls.2016.01358, PMID: 27660633 PMC5014873

[B38] Martins J. T. Rasmussen J. Eriksen J. Arf O. De Notaris C. Moretti L. G. (2022). Biological N fixation activity in soybean can be estimated based on nodule dry weight and is increased by additional inoculation. Rhizosphere 24, 100589. doi: 10.1016/j.rhisph.2022.100589

[B39] Moretti L. G. Crusciol C. A. C. Bossolani J. W. Calonego J. C. Moreira A. Garcia A. . (2021). Beneficial microbial species and metabolites alleviate soybean oxidative damage and increase grain yield during short dry spells. Eur. J. Agron. 127, 126293. doi: 10.1016/j.eja.2021.126293

[B40] Moretti L. G. Crusciol C. A. C. Bossolani J. W. Momesso L. Garcia A. Kuramae E. E. . (2020a). Bacterial consortium and microbial metabolites increase grain quality and soybean yield. J. Soil Sci. Plant Nutr. 20, 1923–1934. doi: 10.1007/s42729-020-00263-5

[B41] Moretti L. G. Crusciol C. A. C. Kuramae E. E. Bossolani J. W. Moreira A. Costa N. R. . (2020b). Effects of growth-promoting bacteria on soybean root activity, plant development, and yield. Agron. J. 112, 418–428. doi: 10.1002/agj2.20010

[B42] Moretti L. G. Crusciol C. A. C. Leite M. F. A. Momesso L. Bossolani J. W. Costa O. Y. A. . (2024). Diverse bacterial consortia: key drivers of rhizosoil fertility modulating microbiome functions, plant physiology, nutrition, and soybean grain yield. Environ. Microbiom. 19, 50. doi: 10.1186/s40793-024-00595-0, PMID: 39030648 PMC11264919

[B43] Moretti L. G. Lazarini E. Bossolani J. W. Parente T. L. Caioni S. Araujo R. S. . (2018). Can additional inoculations increase soybean nodulation and grain yield? Agron. J. 110, 715–721. doi: 10.2134/agronj2017.09.0540

[B44] Niu J. Liu C. Huang M. Liu K. Yan D. (2021). Effects of foliar fertilization: a review of current status and future perspectives. J. Soil Sci. Plant Nutr. 21, 104–118. doi: 10.1007/s42729-020-00346-3

[B45] Nunes F. S. Raimondi A. C. Niedwieski A. C. (2003). Fixação de nitrogênio: estrutura, função e modelagem bioinorgânica das nitrogenases. Quim. Nova. 26, 872–879. doi: 10.1590/S0100-40422003000600016

[B46] O’Hara G. W. Hungria M. Woomer P. Howieson J. G. (2016). “ Counting rhizobia,” in Working with rhizobia. Eds. Howieson J. G. Dilworth M. J. ( Australian Centre for International Agricultural Research, Canberra), 109–124.

[B47] Oliveira S. L. Crusciol C. A. C. Rodrigues V. A. Galeriani T. M. Portugal J. R. Bossolani J. W. . (2022b). Molybdenum foliar fertilization improves photosynthetic metabolism and grain yields of field-grown soybean and maize. Front. Plant Sci. 13. doi: 10.3389/fpls.2022.887682, PMID: 35720532 PMC9199428

[B48] Oliveira J. B. Marques J. P. R. Rodak B. W. Galindo F. S. Carr N. F. Almeida E. . (2022a). Fate of nickel in soybean seeds dressed with different forms of nickel. Rhizosphere 21, 100464. doi: 10.1016/j.rhisph.2021.100464

[B49] Pietrini F. Iori V. Cheremisina A. Shevyakova N. I. Radyukina N. Kuznetsov V. V. . (2015). Evaluation of nickel tolerance in Amaranthus paniculatus L. plants by measuring photosynthesis, oxidative status, antioxidative response and metal-binding molecule content. Environ. Sci. pollut. Res. 22, 482–494. doi: 10.1007/s11356-014-3349-y, PMID: 25081005

[B50] Pinto D. B. B. Ferreira E. Henning F. A. Amaral H. F. Hungria M. Nogueira M. A. (2023). Recovery of Bradyrhizobium cells and effects on the physiological quality of soybean seeds sown in dry soil. J. Seed. Sci. 45, e202345001. doi: 10.1590/2317-1545v45259694

[B51] Rao D. Meng F. Yan X. Zhang M. Yao X. Kim K. S. . (2021). Changes in soil microbial activity, bacterial community composition and function in a long-term continuous soybean cropping system after corn insertion and fertilization. Front. Microbiol. 12, 638326. doi: 10.3389/fmicb.2021.638326, PMID: 33897643 PMC8059791

[B52] Reid C. D. Tissue D. T. Fiscus E. L. Strain B. R. (1997). Comparison of spectrophotometric and radioisotopic methods for the assay of Rubisco in ozone-treated plants. Physiol. Plant 101, 398–404. doi: 10.1111/j.1399-3054.1997.tb01014.x

[B53] Reis A.R.d. de Queiroz Barcelos J. P. de Souza Osório C. R. W. Santos E. F. Lisboa L. A. M. Santini J. M. K. . (2017). A glimpse into the physiological, biochemical and nutritional status of soybean plants under Ni-stress conditions. Environ. Exp. Bot. 144, 76–87. doi: 10.1016/j.envexpbot.2017.10.006

[B54] Rengel Z. (2023). Plant responses to soil-borne ion toxicities. In: Marschner’s Mineral Nutrition of Plants. (Amsterdam, The Netherlands: Elsevier), 665–722. doi: 10.1016/B978-0-12-819773-8.00001-0

[B55] Rodak B. W. Freitas D. S. Bernardes L. F. Lima G. Reis A.R.d. Lavres Junior J. . (2022). Short-term nickel residual effect in field-grown soybeans: nickel-enriched soil acidity amendments promote plant growth and safe soil nickel levels. Arch. Agron. Soil Sci. 68, 1586–1600. doi: 10.1080/03650340.2021.1912325

[B56] Rodak B. W. Freitas D. S. Rossi M. L. Linhares F. S. Moro E. Campos C. N. S. . (2024). A study on nickel application methods for optimizing soybean growth. Sci. Rep. 14, 10556. doi: 10.1038/s41598-024-58149-w, PMID: 38719847 PMC11078936

[B57] Rodrigues T. F. Bender F. R. Sanzovo A. W. S. Ferreira E. Nogueira M. A. Hungria M. (2020). Impact of pesticides in properties of Bradyrhizobium spp. and in the symbiotic performance with soybean. World J. Microbiol. Biotechnol. 36, 172. doi: 10.1007/s11274-020-02949-5, PMID: 33068168

[B58] Santos M. S. Rodrigues T. F. Ferreira E. Megias M. Nogueira M. A. Hungria M. (2020). Method for recovering and counting viable cells from maize seeds inoculated with azospirillum brasilense. J. Pure Appl. Microbiol. 14, 195–204. doi: 10.22207/JPAM.14.1.21

[B59] Sartori F. F. Dieminger Engroff T. Godoy Sanches T. H. Soave J. M. Victório Pessotto M. Felisberto G. . (2023). Potentially harmful effects of seed treatment and pre-inoculation on soybean biological nitrogen fixation and yield. Eur. J. Agron. 142, 126660. doi: 10.1016/j.eja.2022.126660

[B60] Shapiro S. S. Wilk M. B. (1965). An analysis of variance test for normality (Complete Samples). Biometrika 52, 591. doi: 10.2307/2333709

[B61] Shen N. Wang T. Gan Q. Liu S. Wang L. Jin B. (2022). Plant flavonoids: Classification, distribution, biosynthesis, and antioxidant activity. Food Chem. 383, 132531. doi: 10.1016/j.foodchem.2022.132531, PMID: 35413752

[B62] Soil Survey Staff (2014) (Washington, DC, USA: USDA - Natural Resources Conservation Service). doi: 10.1007/978-1-4020-3995-9_269

[B63] Sun W. Fleisher D. Timlin D. Ray C. Wang Z. Beegum S. . (2024). Simulating climate change effects on soil carbon dynamics in a soybean–maize ecosystem: Using improved CO2 emission and transport models. Eur. J. Agron. 159, 127226. doi: 10.1016/j.eja.2024.127226

[B64] Todd C. D. Tipton P. A. Blevins D. G. Piedras P. Pineda M. Polacco J. C. (2006). Update on ureide degradation in legumes. J. Exp. Bot. 57, 5–12. doi: 10.1093/jxb/erj013, PMID: 16317038

[B65] Ureta A.-C. Imperial J. Ruiz-Argüeso T. Palacios J. M. (2005). Rhizobium leguminosarum biovar viciae symbiotic hydrogenase activity and processing are limited by the level of nickel in agricultural soils. Appl. Environ. Microbiol. 71, 7603–7606. doi: 10.1128/AEM.71.11.7603-7606.2005, PMID: 16269813 PMC1287657

[B66] van Raij. B. Cantarella H. Quaggio J. A. Furlani A. M. C. (1997). Recomendações de adubação e calagem para o estado de São Paulo. 2nd. rev (Campinas, Brazil: Fundação IAC Campinas).

[B67] Yusuf M. Fariduddin Q. Hayat S. Ahmad A. (2011). Nickel: an overview of uptake, essentiality and toxicity in plants. Bull. Environ. Contam. Toxicol. 86, 1–17. doi: 10.1007/s00128-010-0171-1, PMID: 21170705

[B68] Zapata D. Rajan N. Mowrer J. Casey K. Schnell R. Hons F. (2021). Long-term tillage effect on with-in season variations in soil conditions and respiration from dryland winter wheat and soybean cropping systems. Sci. Rep. 11, 2344. doi: 10.1038/s41598-021-80979-1, PMID: 33504825 PMC7840680

[B69] Zilli J. É. Pacheco R. S. Gianluppi V. Smiderle O. J. Urquiaga S. Hungria M. (2021). Biological N2 fixation and yield performance of soybean inoculated with Bradyrhizobium. Nutr. Cycl. Agroecosyst. 119, 323–336. doi: 10.1007/s10705-021-10128-7

