# Supplementary material for: Nickel treatment of soybean seeds: evaluating optimal levels for Bradyrhizobium spp. survival, nitrogen fixation, physiological traits and grain yield
Source: Front Plant Sci. 2026 Jan 12;16:1656956. doi: 10.3389/fpls.2025.1656956 (PMC12832769; doi:10.3389/fpls.2025.1656956)
Supplement: Supplementary Table 1 — Experimental information at the three study locations. Cropping seasons 2022/23 and 2023/24. [file DataSheet1.pdf]

SUPPLEMENTARY TABLES AND FIGURES

Supplementary Table 1. Experimental information at the three study locations. Cropping seasons 2022/23 and 2023/24.

Information		Sengés─PR	Botucatu─SP	Selvíria─MS
Geographic Coordinates		24°04'01.2"S 49°34'27.9"W	22°49'30.0"S 48°25'40.7"W	20°20'42.4"S 51°23'50.7"W
Altitude		580 m	783 m	423 m
Production System		No-Tillage		
Row Space		0.50 m		
Cultivar		HO Iguaçu IPRO	NEO 610 IPRO	HO Jacutinga IPRO
Seed Density (m^{-1})		13.1	12.3	14.5
Base Fertilization	2022/23	40 kg P ha^{-1} and 58 kg K ha^{-1}		
	2023/24			
Growing	2022/23	15 Oct. 2022	09 Oct. 2022	20 Oct. 2022
	2023/24	27 Oct. 2023	20 Oct. 2023	23 Oct. 2023
Sampling	2022/23	21 Dec. 2022	15 Dec. 2022	26 Dec. 2022
	2023/24	27 Dec. 2023	20 Dec. 2023	29 Dec. 2023
Harvest	2022/23	03 Mar. 2023	24 Feb. 2023	28 Feb. 2023
	2023/24	10 Mar. 2024	01 Mar. 2024	06 Mar. 2024

Supplementary Table 2. Initial chemical, physical, and biological soil properties at a depth of 0-20 cm and edaphoclimatic conditions at the three study locations.

**Soil Classification**	Unit	Sengés─PR	Botucatu─SP	Selvíria─MS
		Typic Haplorthox		
**Climate Classification**		Cfb	Cfa	Aw
**Chemical Properties**				
pH	$CaCl_2$	5.9	5.8	5.5
Soil Organic Matter	g dm^{-3}	28	24	20
Phosphorus	mg dm^{-3}	36	31	27
Sulfur	mg dm^{-3}	10	11	9
Aluminum	$mmol_c$ dm^{-3}	0	0	3
Potential Acidity	$mmol_c$ dm^{-3}	41	32	24
Potassium	$mmol_c$ dm^{-3}	3.6	3.9	2.1
Calcium	$mmol_c$ dm^{-3}	42	35	21
Magnesium	$mmol_c$ dm^{-3}	20	15	12
Sum of Bases - SB	$mmol_c$ dm^{-3}	65.6	53.9	35.1
Cation Ex. Capacity	$mmol_c$ dm^{-3}	106.6	85.9	59.1
Base Saturation	%	62	63	60
Aluminum Saturation	%	0	0	7.9
Iron	mg dm^{-3}	33	22	19
Copper	mg dm^{-3}	3.4	2.8	1.9
Manganese	mg dm^{-3}	21	15	9
Zinc	mg dm^{-3}	5.9	2.1	4.3
Boron	mg dm^{-3}	0.43	0.39	0.31
Nickel	mg dm^{-3}	0.6	0.5	0.4
**Physical Properties**				
Sand	g dm^{-3}	266	117	556
Silt	g dm^{-3}	148	281	110
Clay	g dm^{-3}	586	502	334
Soil Density	g dm^{-3}	1.20	1.19	1.13
**Biological Properties**				
NMPc	CFU g^{-1}	5.1×10^4	4.2×10^3	5.6×10^3

Supplementary Table 3. Macronutrient concentrations (nitrogen – N; phosphorus – P; potassium – K; calcium – Ca; magnesium – Mg; and sulfur – S) in diagnostic soybean leaves plus petioles at the R_2 phenological stage as a function of nickel (Ni) seed treatment dose. 2022/23 and 2023/24 growing seasons.

**TREATMENT** mg Ni kg^{-1}	**Sengés - PR**											
	N		P		K		Ca		Mg		S	
	g kg^{-1}											
	2022/23	2023/24	2022/23	2023/24	2022/23	2023/24	2022/23	2023/24	2022/23	2023/24	2022/23	2023/24
0	43	45	3.4	3.6	21	23	11	7.4	3.6	3.3	2.4	2.4
60	44	46	3.4	3.8	20	23	11	8.2	3.4	3.6	2.3	2.5
120	42	46	3.7	3.7	22	22	11	7.8	3.7	3.7	2.4	2.4
180	44	46	3.6	3.9	20	23	11	7.6	3.6	3.4	2.5	2.3
240	42	46	3.6	3.5	22	24	10	7.7	3.3	3.3	2.4	2.4
300	42	46	3.6	3.7	21	24	10	7.8	3.4	3.1	2.5	2.5
Test F												
p-value	0.569	0.990	0.144	0.391	0.537	0.382	0.402	0.192	0.357	0.140	0.582	0.873
C.V. (%)	4.9	5.1	4.0	7.3	6.8	5.2	5.4	5.4	7.8	8.1	6.9	11.2
**Botucatu – SP**												
0	43	43	3.0	3.6	22	23	11	7.6	4.0	3.4	2.5	2.4
60	43	45	3.0	3.6	20	24	10	8.2	3.5	3.5	2.5	2.4
120	42	45	3.3	3.6	22	26	10	8.0	3.7	3.7	2.5	2.2
180	44	42	3.3	3.8	19	24	9.7	8.2	3.5	3.8	2.6	2.6
240	43	43	3.2	3.6	20	26	9.9	7.6	3.3	3.7	2.4	2.3
300	41	43	3.2	3.4	19	27	9.3	8.8	3.5	3.9	2.6	2.5
Test F												
p-value	0.937	0.255	0.138	0.583	0.428	0.288	0.265	0.182	0.520	0.220	0.567	0.210
C.V. (%)	9.6	4.8	7.5	8.2	11.2	10.3	8.7	8.0	13.8	8.2	7.0	7.9
**Selvíria - MS**												
0	43	44	3.8	3.6	20	23	11	7.5	3.2	3.4	2.2	2.4
60	45	46	3.8	3.7	21	23	12	8.2	3.3	3.6	2.1	2.5
120	41	46	4.0	3.7	22	24	11	7.9	3.6	3.7	2.3	2.3
180	44	44	3.9	3.8	22	24	11	7.9	3.7	3.6	2.4	2.4
240	42	45	4.0	3.5	23	25	11	7.6	3.4	3.5	2.4	2.4
300	42	44	3.9	3.5	22	25	11	8.3	3.3	3.5	2.4	2.5
Test F												
p-value	0.460	0.523	0.621	0.106	0.159	0.112	0.803	0.116	0.272	0.596	0.548	0.589
C.V. (%)	6.2	3.6	5.7	4.3	7.7	4.6	9.4	5.3	7.1	6.6	9.2	5.6

*Data were analyzed by analysis of variance (ANOVA).

Supplementary Table 4. Micronutrient concentrations (iron – Fe; copper – Cu; zinc – Zn; manganese – Mn; boron – B; and nickel – Ni) in diagnostic soybean leaves plus petioles at the R_2 phenological stage as a function of nickel (Ni) seed treatment dose. 2022/23 and 2023/24 growing seasons.

TREATMENT mg Ni kg^{-1}	Fe		Cu		Zn		Mn		B		Ni	
	mg kg^{-1}											
	2022/23	2023/24	2022/23	2023/24	2022/23	2023/24	2022/23	2023/24	2022/23	2023/24	2022/23	2023/24
**Sengés - PR**												
0	112	92	11	13	47	37	68	52	48	47	0.6	0.5
60	119	92	11	13	50	39	64	52	48	48	0.6	0.4
120	122	94	11	14	47	40	65	53	47	49	0.6	0.4
180	123	96	11	12	50	40	72	55	48	50	0.5	0.5
240	120	99	11	13	46	41	67	57	48	56	0.5	0.5
300	115	100	11	12	47	44	68	60	47	53	0.5	0.6
Test F												
p-value	0.465	0.131	0.544	0.417	0.591	0.204	0.244	0.149	0.992	0.343	0.311	0.342
C.V. (%)	7.1	5.4	6.3	13.6	7.5	8.5	6.4	7.3	6.1	12.6	24.8	10.0
**Botucatu – SP**												
0	108	101	11	13	48	44	80	62	46	51	0.4	0.6
60	107	104	9.8	12	51	44	73	61	47	52	0.6	0.6
120	109	108	10	13	48	46	69	62	44	54	0.6	0.6
180	108	99	11	13	51	44	77	61	46	50	0.5	0.6
240	109	101	10	13	52	46	76	62	47	54	0.6	0.6
300	108	101	9.3	13	52	44	74	60	45	53	0.6	0.6
Test F												
p-value	0.999	0.853	0.227	0.696	0.729	0.798	0.491	0.857	0.553	0.619	0.376	0.971
C.V. (%)	10.7	10.5	8.6	11.0	9.6	7.9	10.1	5.4	5.6	6.5	23.6	15.3
**Selvíria – MS**												
0	127	96	11	13	47	40	56	62	50	49	0.5	0.6
60	132	98	11	12	49	41	56	60	49	50	0.6	0.5
120	135	101	12	14	46	42	60	61	50	51	0.6	0.5
180	138	98	12	12	51	42	63	60	49	50	0.6	0.5
240	130	100	11	13	40	43	60	62	48	55	0.6	0.6
300	122	101	12	13	43	44	62	60	49	53	0.6	0.6
Test F												
p-value	0.166	0.818	0.280	0.477	0.279	0.597	0.195	0.857	0.987	0.202	0.274	0.352
C.V. (%)	7.9	6.3	7.3	9.4	13.9	6.9	10.2	5.4	9.2	6.8	17.3	10.8

*Data were analyzed by analysis of variance (ANOVA).

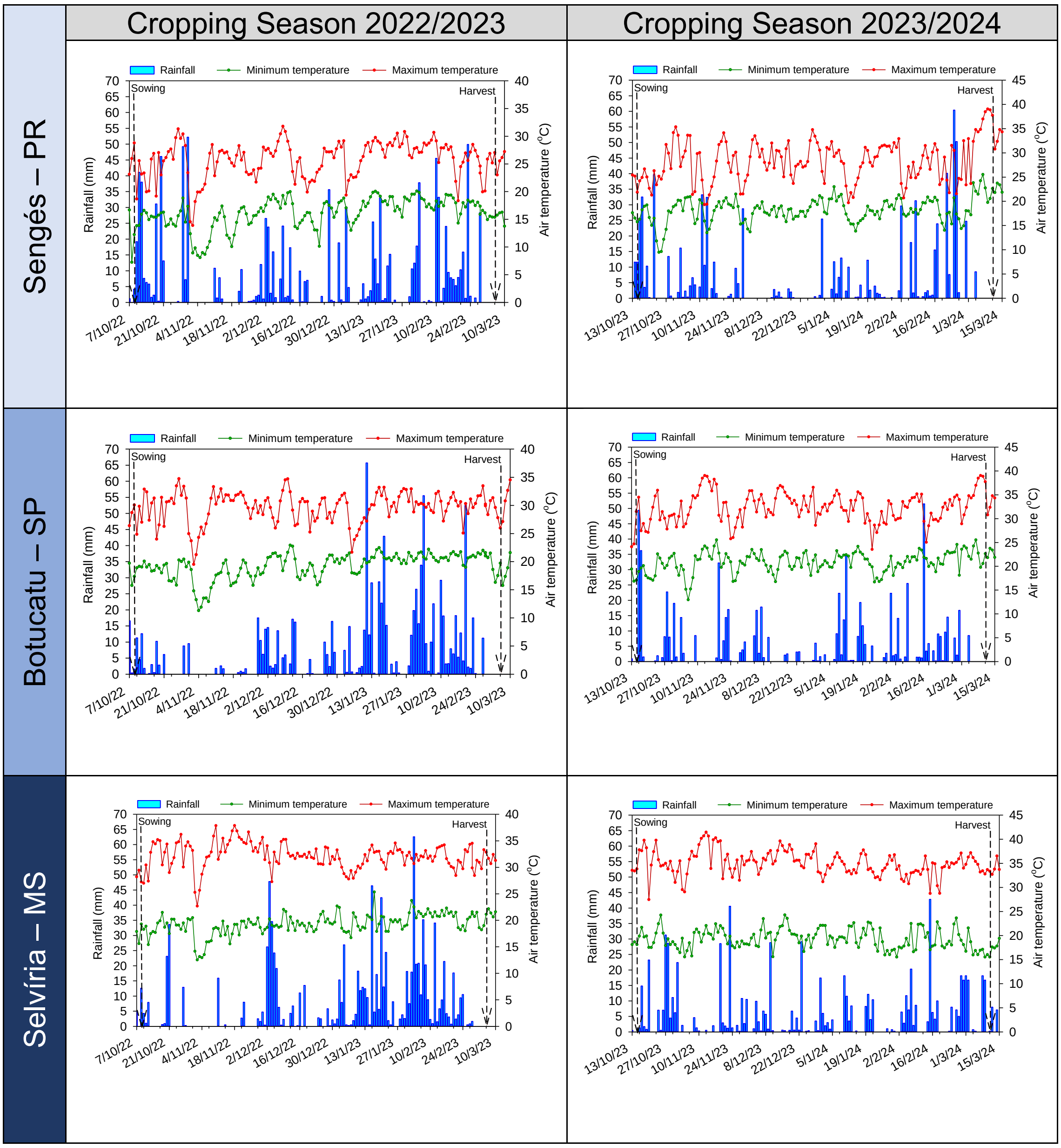

Supplementary Figure 1. Average biweekly temperatures and precipitation (mm) during the first and second growing seasons in Sengés, Botucatu, and Selvíria. Cropping seasons 2022/23 and 2023/24.
